# OpenAI's ‘Deep Research’ for the Generation of Comprehensive Referenced Medical Text: Uses and Cautions

**DOI:** 10.1111/ans.70161

**Published:** 2025-05-05

**Authors:** Daniel Jesudason, Christina Gao, Ishith Seth, Weng Onn Chan, Stephen Bacchi

**Affiliations:** ^1^ Faculty of Health & Medical Sciences The University of Adelaide Adelaide South Australia Australia; ^2^ Department of Plastic & Reconstructive Surgery Peninsula Health Frankston Victoria Australia; ^3^ The Queen Elizabeth Hospital Woodville South South Australia Australia; ^4^ Harvard Medical School Harvard University Boston Massachusetts USA; ^5^ Lyell McEwin Hospital Adelaide South Australia Australia

**Keywords:** artificial intelligence, deep research, large language models, machine learning, medical text

Surgical research has become an area of great productivity in Australia and New Zealand, with many pre‐eminent and prospective surgeons electing to engage in research projects alongside their clinical duties [1]. These research endeavours contribute greatly to the betterment of the surgical sciences and the dissemination of evidence‐based medicine [2]. Research advances the surgical vocation by driving the innovation, refinement, and optimisation of surgical procedures and guidelines that dictate contemporary standards of practice [3]. One evolving area of surgical research pertains to the application of artificial intelligence (AI) to streamline, enhance, and accelerate various aspects of the surgical craft.

In February 2025, OpenAI (the developers of ChatGPT) released a new platform called Deep Research (DR) [4]. The AI platform was designed to rapidly access and analyse vast datasets of literature, synthesise their findings, and then generate a written output in the desired format, language, and complexity of the user. DR presents an opportunity to bridge the gap between the ever‐expanding reservoir of medical knowledge and the surgeon's ability to comprehend and apply it. However, the system poses certain risks that, if left unaddressed, have the potential to compromise the integrity of surgical scholarship and undermine the very foundations of evidence‐based medicine and clinical decision‐making. In this way, we believe that a balanced approach to this new platform is required.

DR, which can be found under the ‘Explore GPTs’ panel of ChatGPT, is advertised as an ‘AI‐powered research assistant’. It allows a user to input a research topic and ask several clarifying questions, after which it will generate a written output several pages in length. The platform is already attracting significant attention in the scientific community due to its ability to produce referenced text [5]. For instance, we used DR to generate written pieces on two potentially controversial evidence‐based medicine topics; antithrombotic therapy for carotid dissection, and surgical and transcatheter aortic valve replacement for complex asymptomatic aortic stenosis (Supporting Information S1 and S2). The output remains non‐deterministic, with identical prompts producing differing results.

The quality of these outputs is shown in circumstances beyond controversial evidence‐based topics. Potential utility could be envisioned in the summarisation of institutional websites to create patient handouts, and synthesising information on questions for topics appropriate for trainee education (Supporting Information S3). Such comprehensive referenced outputs also have potential utility in the preparation of grant applications and other related research documents, such as ethics applications and project proposals. Thus, the prospective benefit to the surgical community is significant.

However, while DR presents several advantages to the broader surgical community, its application in research has many potential limitations, which may complicate surgical education, decision‐making, and subsequent patient care. A large proportion of evidence‐based surgery revolves around the ability to critically appraise and synthesise information [6]. However, generative AI systems present users with information that has been pre‐synthesised, already featuring the elimination of unnecessary data and having made preformed conclusions. In this way, there is a legitimate concern that future generations of researchers may become overly reliant on algorithmic outputs, leading to a decline in their ability to independently assess the validity, methodology, and applicability of primary studies. This can be considered analogous to the concerns that intraoperative robotic‐assisted surgery would result in the erosion of core surgical skills [7].

Another major concern is the possibility of algorithmic bias in AI systems, especially given their proprietary and often opaque nature. This is especially relevant in medical and surgical research contexts, as clinical researchers may only possess a rudimentary understanding of the processes that underpin AI algorithms, if that. It is therefore extremely difficult for the reader to determine factors such as the inclusion or exclusion criteria of a published review, the processes used in the evaluation of conflicting evidence, and implicit biases embedded within the AI model's decision‐making. If DR selectively filters results based on unknown or undisclosed criteria, there is a significant risk that research, and hence real‐world surgical guidelines, may become skewed towards certain viewpoints, methodologies, or institutional biases, which may not be immediately apparent to a surgical readership. Thus, platforms like DR may contribute to a false sense of objectivity within the evidence appraisal process, promoting certain narratives that influence surgical practice in potentially detrimental ways.

One particularly important source of algorithmic bias to consider is DR's inability to access content behind paywalls [8]. If AI platforms can only analyse open‐access literature, the synthesised body of evidence may be disproportionately skewed towards a select few studies and guidelines, which may not necessarily represent the highest quality of current evidence in surgical research. Many high‐impact surgical journals operate behind paywalls, meaning their articles may be excluded from AI‐generated analyses. The possible implications of these biases are significant. Many landmark clinical trials and reviews are published in subscription‐based journals, yet could, under an AI‐driven research methodology, be buried beneath pre‐prints, lower‐impact publications, or repositories with more relaxed peer‐review processes. Thus, there is a genuine danger that outputs on surgical topics could be shaped by an incomplete dataset.

As AI‐driven systems continue to be used for research, the issue of iterative use becomes apparent. If AI‐generated content is repeatedly fed into subsequent AI reviews (meaning AI summarises AI), the content becomes subject to a progressive distortion of original evidence, creating an ecosystem where the verification of published outputs becomes increasingly difficult. The incapability to clearly identify human versus AI‐generated research could thus perpetuate a self‐reinforcing cycle, where AI‐derived insights are assumed to be authoritative without any method of verification.

Therefore, while novel AI platforms such as DR offer unprecedented opportunities to enhance surgical research by streamlining medical literature synthesis, they also introduce significant risks that cannot be overlooked. As AI continues to pave its path in the modern surgical space, the surgical community must treat its integration with a careful balance of cautious optimism and rigorous oversight. To truly derive the maximum benefit from this emerging technology, we must look to leverage its numerous strengths and ultimately seek to increase productivity without compromising the integrity and autonomy of independent human thought.

## Author Contributions

**Daniel Jesudason:** writing – original draft, writing – review and editing. **Christina Gao:** writing – review and editing. **Ishith Seth:** writing – review and editing. **Weng Onn Chan:** writing – review and editing, supervision. **Stephen Bacchi:** writing – original draft, supervision, writing – review and editing, conceptualisation.

## Conflicts of Interest

The authors declare no conflicts of interest.

## Supporting information


Data S1.

Data S2.

Data S3.

## Data Availability

The authors have nothing to report.
