# Supplementary material for: OpenAI's ‘Deep Research’ for the Generation of Comprehensive Referenced Medical Text: Uses and Cautions
Source: ANZ J Surg. 2025 May 5;95(11):2250–2. doi: 10.1111/ans.70161 (PMC12641354; doi:10.1111/ans.70161)
Supplement: Supplementary file 1 — Data S1. [file ANS-95-2250-s002.pdf]

Carotid Artery Dissection Management

Carotid artery dissection management. In particular, I am interested in the evidence and guidelines underpinning the selection of different antiplatelets (such as aspirin, clopidogrel, and ticagrelor) and anticoagulants (such as enoxaparin) and the combination of these agents. I am also interested in the duration of therapy with these agents and schedules for follow up imaging

To provide the most relevant evidence and guidelines on the management of carotid artery dissection, could you specify:

- Are you looking for recommendations based on specific patient populations (e.g., ischemic stroke, trauma-induced dissection, spontaneous dissection)?
- Would you like comparisons of different antiplatelet and anticoagulant agents in terms of efficacy and safety?
- Are you interested in both acute and long-term management, including duration of therapy and follow-up imaging intervals?
- Do you have a preference for specific guideline sources (e.g., AHA/ASA, ESC, NICE)?

This will help me tailor the research to your needs.

Good questions, I'll answer point-by-point:

Are you looking for recommendations based on specific patient populations (e.g., ischemic stroke, trauma-induced dissection, spontaneous dissection)?

All.

Would you like comparisons of different antiplatelet and anticoagulant agents in terms of efficacy and safety?

Yes.

Are you interested in both acute and long-term management, including duration of therapy and follow-up imaging intervals?

Yes.

Do you have a preference for specific guideline sources (e.g., AHA/ASA, ESC, NICE)?

No specific preference.

Got it. I'll gather evidence and guidelines on the management of carotid artery dissection, covering both ischemic stroke, trauma-induced, and spontaneous cases. The research will compare different antiplatelet (aspirin, clopidogrel, ticagrelor) and anticoagulant agents (including enoxaparin) in terms of efficacy and safety. I'll also look into recommendations for the duration of therapy and schedules for follow-up imaging from major guidelines such as AHA/ASA, ESC, NICE, and other relevant sources. I'll update you once I have the findings.

Carotid Artery Dissection – Management Overview

Carotid artery dissection (tear in the arterial wall) can occur spontaneously or after trauma, and it is a significant cause of ischemic stroke in younger patients. Management focuses on preventing stroke by antithrombotic therapy, with decisions tailored to each case. Below we compare antiplatelet vs anticoagulant strategies (including specific agents like aspirin, clopidogrel, ticagrelor, and enoxaparin) in terms of efficacy, safety, and guidelines. We also outline recommendations for therapy duration and follow-up imaging, drawing on major guidelines (AHA/ASA, ESO, NICE) and recent studies.

Acute Stroke in Carotid Dissection

When a carotid dissection causes an acute ischemic stroke, initial management follows standard stroke protocols. **Intravenous thrombolysis** (alteplase) is recommended if within the 4.5-hour window and no contraindications, even in the setting of dissection ESO-STROKE.ORG AHAJOURNALS.ORG. Similarly, **mechanical thrombectomy** is indicated for large-vessel occlusions (e.g. carotid T/L occlusions) in otherwise eligible patients ESO-STROKE.ORG AHAJOURNALS.ORG. These acute interventions should not be withheld solely due to the dissection. Once acute therapy is addressed, attention turns to antithrombotic prevention of further ischemia.

Antiplatelet vs Anticoagulant Therapy: Efficacy and Safety

Guideline consensus: Both antiplatelet (AP) and anticoagulant (AC) therapies are accepted options for cervical artery dissection. Guidelines uniformly state that either can be used, as no high-quality evidence clearly favors one over the other for preventing stroke in extracranial dissection ESO-STROKE.ORG NICE.ORG.UK. For example, the European Stroke Organisation (ESO) 2021 guideline gives a strong recommendation that **either anticoagulants or antiplatelets may be prescribed in acute extracranial dissection** ESO-STROKE.ORG. The UK NICE guideline likewise advises offering *either* anticoagulation or antiplatelet agents for stroke secondary to arterial dissection NICE.ORG.UK. An American Heart Association (AHA) scientific statement (2024) notes no overall superiority of one strategy and urges individualizing the choice based on patient factors (ischemic risk vs. bleeding risk) AHAJOURNALS.ORG.

Efficacy: Multiple studies have compared outcomes with antiplatelets vs anticoagulation. The landmark CADISS trial (2015) found no significant difference in stroke recurrence between anticoagulation (heparin/warfarin) and antiplatelet therapy in cervical dissections ESO-STROKE.ORG. More recently, a large meta-analysis of 11 studies (including 2 RCTs, total N≈5000) indicated anticoagulation may reduce the risk of recurrent ischemic stroke by ~37% relative (RR ~0.63) compared to antiplatelets AHAJOURNALS.ORG. However, this **potential efficacy benefit** comes at the cost of higher bleeding risk. The same meta-analysis showed anticoagulation was associated with roughly **double the risk of major hemorrhage** (RR ~2.25) versus antiplatelet therapy AHAJOURNALS.ORG. Importantly, there was *no significant difference in mortality or intracranial hemorrhagic stroke* between the two strategies AHAJOURNALS.ORG. In summary, anticoagulants might slightly lower recurrent stroke odds, but any gain is offset by increased bleeding, so overall outcomes are similar. This trade-off underlies why guidelines deem both strategies reasonable, tailoring the choice to the individual.

Safety: Antiplatelet therapy (e.g. aspirin) generally carries a lower risk of serious bleeding than full anticoagulation. Anticoagulants (heparin, warfarin, or DOACs) pose higher risk of systemic bleeding and hemorrhagic conversion if a large infarction is present. Thus, patient factors guide selection:

- If ischemic risk is judged very high (e.g. an occlusive dissection with intraluminal thrombus) and hemorrhagic risk low, clinicians may lean toward anticoagulation for more potent thrombus prevention AHAJOURNALS.ORG. For instance, the AHA notes it's

reasonable to use **heparin bridging to warfarin** in patients with high stroke risk features (intraluminal clot, critical stenosis) **and low intracranial hemorrhage risk** (no large infarct, no intracranial extension of dissection) AHAJOURNALS.ORG .

- Conversely, if bleed risk is higher – e.g. large infarct present, uncontrolled hypertension, or extension of the dissection into an intracranial segment – an antiplatelet may be safer. Notably, **intracranial artery dissections** (inside the skull) are often managed with antiplatelets over anticoagulants, because an intracranial dissection can cause subarachnoid hemorrhage; expert consensus favors avoiding anticoagulation in this scenario ESO-STROKE.ORG .

Overall, current evidence does not show a dramatic difference in efficacy between antiplatelets and anticoagulants for carotid dissection, so safety and patient-specific factors (including trauma context, bleeding risk, and patient preferences) drive the decision AHAJOURNALS.ORG . This approach applies to **spontaneous and traumatic dissections** alike – both causes create similar ischemic risks. Trauma-induced dissections should be treated with antithrombotic therapy once any bleeding injuries are stabilized. In trauma patients with multiple injuries, antiplatelet therapy might be chosen initially if full anticoagulation is contraindicated, but if no such contraindications exist, either therapy is appropriate ESO-STROKE.ORG .

Antiplatelet Therapy Options (Aspirin, Clopidogrel, Ticagrelor)

For carotid dissections managed with antiplatelet therapy, **aspirin** (usually 300 mg loading, then 75–100 mg daily) is a standard first-line agent. **Clopidogrel** (75 mg daily, after a loading dose) is an alternative monotherapy, particularly if aspirin intolerance or as part of combination therapy. There is no direct trial comparing specific antiplatelet drugs in dissection, but in general stroke prevention, clopidogrel is about equally effective to aspirin (with some evidence of slight superiority in certain populations). **Ticagrelor**, a more potent P2Y12 inhibitor, has been studied in acute stroke/TIA settings (but not specifically in dissection). In the THALES trial (2020) involving minor strokes/TIAs (mostly atherosclerotic origin), adding ticagrelor to aspirin for 30 days reduced the risk of stroke or death by ~17% relative to aspirin alone, at the expense of increased risk of severe hemorrhage AHAJOURNALS.ORG . Thus, ticagrelor can be considered in certain high-risk patients or if clopidogrel

is unsuitable, but it tends to cause more bleeding.

Monotherapy vs dual antiplatelet therapy (DAPT): For most cervical artery dissection patients, a **single antiplatelet agent** is used. However, short-term dual therapy (typically aspirin + clopidogrel) can be considered in the acute phase for select patients. The rationale comes from trials in non-dissection strokes (CHANCE, POINT) showing DAPT for 21–90 days improves outcomes in high-risk TIA or minor stroke ESO-STROKE.ORG. The AHA scientific statement (2024) suggests that although specific evidence in dissection is limited, a **short course of DAPT (aspirin plus clopidogrel)** with an appropriate loading dose *"might be preferred over monotherapy"* in the first few weeks **for patients who present with a minor stroke or TIA** (i.e. those who would have qualified for the dual antiplatelet trials) AHAJOURNALS.ORG. In practice, some clinicians give aspirin + clopidogrel for the initial 2–4 weeks after a dissection-related TIA/minor stroke, then de-escalate to single antiplatelet. This strategy aims to reduce early recurrent embolism while balancing bleeding risk. DAPT is **not** continued long-term, since bleeding risk rises over time; after the acute period, patients are maintained on monotherapy AHAJOURNALS.ORG. Ticagrelor can substitute for clopidogrel in a dual regimen if needed (as studied in THALES), but routine use of aspirin–ticagrelor dual therapy is reserved for specific cases given bleeding considerations.

In summary, all three antiplatelet agents (aspirin, clopidogrel, ticagrelor) are viable, with **aspirin or clopidogrel monotherapy** being first-line choices. No strong evidence favors one over the other in dissection, so choice often depends on patient factors (allergies, tolerance, clinical scenario). **Dual antiplatelet therapy** is an option for the early period after a dissection-related TIA or small stroke, to maximize protection against recurrent events, but it should be limited to a short duration (e.g. 2–4 weeks) AHAJOURNALS.ORG.

Anticoagulation Options (Heparin, Enoxaparin, Warfarin, DOACs)

If anticoagulation is chosen, therapy typically begins with **rapid-acting agents** followed by an oral anticoagulant:

- **Intravenous unfractionated heparin or subcutaneous low-molecular-weight heparin** (e.g. enoxaparin) is often initiated in the acute phase. These provide immediate anticoagulation and can be quickly adjusted or reversed if needed. Enoxaparin is commonly used for convenience and reliable dosing.
- This is usually **bridged to a Vitamin K antagonist (VKA)** such as warfarin, with a target INR ~2.0–3.0 PORTAILVASCULAIRE.FR. Warfarin is then continued for the intended duration. (In the CADISS and TREAT-CAD trials, most patients in the AC arm received heparin→warfarin).
- **Direct oral anticoagulants (DOACs)** (e.g. dabigatran, rivaroxaban, apixaban) are an emerging option. While DOACs are not yet backed by RCT evidence specifically in carotid dissection, they are increasingly used in practice as an alternative to warfarin. An observational analysis found similar rates of stroke/TIA recurrence in patients treated with DOACs versus warfarin for cervical dissections PORTAILVASCULAIRE.FR. DOACs have the practical advantages of fixed dosing and lower intracranial bleed risk (demonstrated in other conditions like AF), but again, no randomized data in dissection to formally recommend them. Guidelines note the lack of evidence for DOACs in this setting PORTAILVASCULAIRE.FR, so their use is based on clinician judgment.

Safety considerations: If a patient has had a large territorial stroke from the dissection, clinicians often delay or avoid starting full-dose anticoagulation for ~1–2 weeks, using aspirin instead in the interim, due to the risk of hemorrhagic transformation of the infarct. In patients with only mild symptoms or TIA, immediate anticoagulation is more feasible. Trauma patients with solid-organ injury or neurosurgical concerns might also preclude heparin/warfarin – in those cases, antiplatelet therapy is a safer initial choice until bleeding risk subsides.

Notably, combining an anticoagulant and an antiplatelet (concurrent) is **not routinely recommended** in carotid dissection due to high bleeding risk, unless there is a separate indication (for example, if the patient has a coronary stent or other compelling reason). The strategies are generally **anticoagulation alone** *or* **antiplatelet alone**, not both simultaneously.

Duration of Therapy

The duration of antithrombotic treatment for carotid dissection is finite, since dissections often heal over time. Guidelines consistently recommend about **3 to 6 months of therapy**, followed by re-assessment:

- The AHA/ASA statement suggests continuing antithrombotic therapy for *at least* **3–6 months** AHAJOURNALS.ORG. After 3–6 months, if the artery has healed or the risk is deemed low, therapy can often be discontinued. Decisions to extend beyond 6 months should be individualized, based on the patient's vascular risk factors and any persistent arterial abnormalities AHAJOURNALS.ORG.
- The typical approach is to treat for **3 months**, then perform follow-up imaging (see below). If the dissection has resolved (normal lumen or only minimal changes), one can consider stopping antithrombotics at 3 months or simplifying to a single antiplatelet (if, for instance, the patient has other atherosclerotic risk factors). Some clinicians continue out to **6 months** of therapy regardless, especially if there were significant ischemic events, and then stop if imaging by 6 months shows healing.
- **Extended therapy:** In a minority of cases, antithrombotics are continued beyond 6 months. Examples might be if the dissection has not healed and there is a persistent severe stenosis or large dissecting aneurysm, or if the patient has recurrent symptoms. Even then, continuation needs to be weighed against bleeding risk. AHA notes it's reasonable to extend therapy past 6 months in select cases (taking into account overall risk profile and imaging features) AHAJOURNALS.ORG. In practice, some experts will continue an antiplatelet (rather than anticoagulant) long-term if a residual dissecting aneurysm or stenosis remains, on the rationale that it may afford some protection with less bleeding risk ESO-STROKE.ORG. There is, however, no clear evidence that prolonged treatment beyond 6–12 months reduces late complications in dissection.

Importantly, once antithrombotic therapy is stopped, patients should continue to be managed with **vascular risk reduction** (e.g. control of blood pressure, no smoking, etc.), and advised on **avoiding major cervical trauma** especially in the early healing phase. The risk of a *recurrent dissection* in a new artery is low (roughly 1–2% per year) but is highest in the first few months

AHAJOURNALS.ORG. Therefore, precautions like avoiding high-impact sports or cervical chiropractic manipulations are advised for at least the first 1–6 months after a dissection AHAJOURNALS.ORG. In patients with known connective tissue disorders or multiple recurrent dissections, such risky activities might be avoided indefinitely AHAJOURNALS.ORG.

Follow-Up Imaging and Outcomes

Imaging schedule: Follow-up vascular imaging is crucial to guide management duration. Typically, a repeat imaging study is obtained around **3 months** after the dissection. This can be done with **CTA or MRA**, or sometimes carotid ultrasound for extracranial carotid lesions. Ultrasound is useful for tracking lumen changes over time and is often used at 3 months as a first check

AHAJOURNALS.ORG . If 3-month imaging shows the artery has completely healed or near-normalized, clinicians may opt to stop anticoagulation or switch to a prophylactic antiplatelet. If significant narrowing or an aneurysmal dilatation persists, treatment is usually continued and imaging may be repeated again at **6 months** and/or **12 months**.

Healing rates: Many carotid dissections will spontaneously heal (recanalize) during the first months. Data indicate about **one-third of completely occluded arteries reopen by ~4 months** on average

AHAJOURNALS.ORG . Overall, roughly 50% of dissected carotid arteries show complete healing and others show partial improvement by 3–6 months AHAJOURNALS.ORG . Healing can continue up to **12 months** post-dissection, but beyond 1 year further recanalization is rare AHAJOURNALS.ORG . This timeline informs the typical 3–6 month treatment window.

Crucially, studies show that after ~6 months, the stroke risk is very low even if the artery is still narrowed or occluded. There is **no strong correlation between a persistent residual stenosis and late stroke** beyond the acute phase

AHAJOURNALS.ORG . In other words, if a patient's carotid remains occluded or >70% stenosed chronically, it usually becomes a stable lesion with collateral circulation and does not necessarily cause new strokes after the initial period. This supports stopping aggressive therapy at 6 months in healed or stable cases.

Dissecting aneurysms: Some dissections leave a residual aneurysmal dilation of the artery wall. About 10–20% of patients develop a pseudoaneurysm at the site of dissection

AHAJOURNALS.ORG . The good news is these **dissecting aneurysms typically have a benign prognosis**: they rarely rupture and have not been shown to increase stroke risk AHAJOURNALS.ORG . Most will either remain stable or even shrink over time in about half of cases AHAJOURNALS.ORG . Because of this, conservative management is generally continued. Neither antiplatelet nor

anticoagulant therapy has been found to significantly influence whether an aneurysm resolves or persists AHAJOURNALS.ORG (the choice of antithrombotic is about stroke prevention, not aneurysm healing). If a large dissecting aneurysm is enlarging or compressing adjacent structures, then intervention (such as stent-assisted coiling or flow-diversion) may be considered, but this is rare AHAJOURNALS.ORG .

Surveillance: After discontinuing therapy, a final imaging at 12 months can confirm stable healing. Long-term follow-up beyond a year is not usually needed unless new symptoms occur. Patients should be counseled on stroke symptoms and to report any recurrence of warning signs (neck pain with neuro symptoms).

Summary of Key Recommendations

- **Antithrombotic choice (antiplatelet vs anticoagulant):** Both are acceptable. Current guidelines allow either, as trials have not proven one definitively better ESO-STROKE.ORG NICE.ORG.UK . Anticoagulation (heparin bridged to warfarin or a DOAC) may slightly reduce early ischemic risk but carries more bleeding risk AHAJOURNALS.ORG . Antiplatelet therapy (aspirin, etc.) is simpler and safer in many cases. **Tailor the choice** to the patient's situation – consider anticoagulation if high risk of clotting (e.g. intraluminal thrombus, extensive dissection) and low bleed risk, but use antiplatelet if bleed risk is higher or if the dissection involves intracranial segments (to avoid hemorrhage) AHAJOURNALS.ORG ESO-STROKE.ORG . Both approaches are supported by guidelines (Class I or IIa recommendations) ESO-STROKE.ORG .

- **Specific agents:** Aspirin 75–100 mg daily (after loading) is a first-line antiplatelet; clopidogrel is an equivalent alternative. Ticagrelor may be used in place of clopidogrel in dual therapy or if needed, with awareness of higher bleeding tendency. If anticoagulation is used, start with heparin or enoxaparin, then transition to warfarin (INR 2–3) or consider a DOAC (off-label, emerging practice). **Do not combine** antiplatelet and anticoagulant routinely (avoiding dual therapy except short-term DAPT in select cases).

- **Monotherapy vs combination: Monotherapy** is the standard for both anticoagulants and antiplatelets in dissection. **Dual Antiplatelet Therapy** (aspirin + clopidogrel) is recommended for a *short duration* (up to 90 days, typically 1 month) in patients with minor stroke or TIA from dissection, mirroring general stroke guidelines AHAJOURNALS.ORG. It should be followed by single-agent therapy. There is insufficient data on combining two antiplatelets long-term or on using two antithrombotic classes together AHAJOURNALS.ORG – these are not generally recommended due to bleeding risk.

- **Duration of therapy:** Treat for about **3–6 months** in total AHAJOURNALS.ORG. Perform follow-up imaging at around 3 months. If the artery has healed and the patient is stable, transitioning off therapy at 3–6 months is usually appropriate. If the dissection is not healed or the patient had significant stroke, continue to 6 months and reassess. Extending beyond 6 months is rarely needed except in unusual cases; any extension should be based on persistent arterial abnormalities and overall risk profile AHAJOURNALS.ORG.

- **Follow-up imaging: Always obtain follow-up vascular imaging** (CTA, MRA, or ultrasound) to check vessel status. Many clinicians image at 3 months and again at 6 months if needed. Expect that ~50% of dissected carotids will recanalize by 3–6 months AHAJOURNALS.ORG. Recanalization after 1 year is uncommon AHAJOURNALS.ORG. If a residual dissection or aneurysm is present but stable at 6–12 months, ongoing surveillance is optional as long-term stroke risk is low. Dissecting aneurysms usually do not require invasive treatment unless causing symptoms AHAJOURNALS.ORG.

In conclusion, the management of carotid artery dissection centers on prompt antithrombotic therapy (antiplatelet or anticoagulation) to prevent ischemic stroke, applied for a limited duration (months) while the artery heals. Major guidelines (AHA/ASA, ESO, NICE) concur that either antiplatelets or anticoagulants can be used, so treatment is individualized ESO-STROKE.ORG NICE.ORG.UK. Early combination antiplatelet therapy may be used in minor stroke cases, and thrombolysis/thrombectomy should be utilized in acute stroke when indicated. Careful follow-up with imaging ensures that therapy is appropriately continued or ceased. This balanced approach maximizes stroke prevention while minimizing bleeding, and current evidence supports it as the standard of care in carotid dissection management AHAJOURNALS.ORG AHAJOURNALS.ORG.

Sources:

- AHA/ASA Scientific Statement on Cervical Artery Dissections, 2024 AHAJOURNALS.ORG AHAJOURNALS.ORG AHAJOURNALS.ORG
- European Stroke Organisation (ESO) Guidelines for Extracranial Artery Dissection, 2021 ESO-STROKE.ORG ESO-STROKE.ORG
- NICE Clinical Guideline NG128 (Stroke Management), 2019 NICE.ORG.UK
- L. Yaghi et al., *Stroke* 2024 – Meta-analysis of antiplatelet vs anticoagulation in cervical artery dissection AHAJOURNALS.ORG
- H. S. Markus et al. (CADISS trial), *Lancet Neurol* 2015 – Antithrombotics in carotid dissection ESO-STROKE.ORG
- N. Fischer et al. (TREAT-CAD trial), *Lancet Neurol* 2021 – Aspirin vs VKA in dissection PORTAILVASCULAIRE.FR
- TIA/stroke dual therapy trials: CHANCE, POINT, THALES (for context of combination antiplatelet use) AHAJOURNALS.ORG AHAJOURNALS.ORG
