# Supplementary material for: OpenAI's ‘Deep Research’ for the Generation of Comprehensive Referenced Medical Text: Uses and Cautions
Source: ANZ J Surg. 2025 May 5;95(11):2250–2. doi: 10.1111/ans.70161 (PMC12641354; doi:10.1111/ans.70161)
Supplement: Supplementary file 2 — Data S2. [file ANS-95-2250-s001.pdf]

Aortic Stenosis Intervention Evidence

I am trying to determine whether there is sufficient evidence to support routine intervention (either by transcatheter aortic valve replacement or surgical aortic valve replacement) for aortic stenosis that is severe but asymptomatic.

Are you looking for a review of recent clinical studies and guidelines on this topic, or a summary of expert opinions and meta-analyses? Additionally, do you have a preferred time range for the studies (e.g., last 5 years) and any specific sources you would like prioritized (e.g., American Heart Association, European Society of Cardiology)? Let me know so I can refine the research accordingly.

Both primary research, guidelines, systematic reviews and meta-analyses. When there are conflicting positions I want these highlighted. I have no preference with respect to the source of the information geographically. Limiting the research to the last 5-10 years is reasonable.

Got it! I'll conduct a comprehensive review of primary research studies, clinical guidelines, systematic reviews, and meta-analyses from the last 5-10 years regarding routine intervention for severe but asymptomatic aortic stenosis. I'll also highlight conflicting positions where they exist. I'll get back to you with the findings soon.

Routine Intervention for Severe Asymptomatic Aortic Stenosis: Evidence Review

Background and Clinical Question

Severe aortic stenosis (AS) is a critical valve narrowing that, when symptomatic, warrants prompt aortic valve replacement (either surgical AVR or transcatheter TAVR). However, in *asymptomatic* patients with severe AS, the optimal timing of intervention is debated. Historically, guidelines favored "watchful waiting" until symptom onset or left ventricular (LV) dysfunction, to avoid surgical risks in patients who feel well. The concern is that unnecessary early intervention could expose patients to procedural risk without clear benefit, whereas delayed intervention risks sudden death or irreversible cardiac damage. This review examines recent evidence (last 5–10 years) on whether early AVR (TAVR or SAVR) in asymptomatic severe AS improves outcomes compared to conservative observation, highlighting key studies, guidelines, meta-analyses, and areas of controversy.

Guideline Recommendations (ACC/AHA vs ESC)

ACC/AHA (U.S.) Guidelines (2020): The American Heart Association/American College of Cardiology guidelines continue to prioritize symptom status and ventricular function for timing of AVR

TCTMD.COM. In asymptomatic severe AS, a Class I indication for AVR exists **only if** there is evidence of LV systolic dysfunction (LVEF <50%) or if the patient is undergoing other cardiac surgery (e.g. CABG) TCTMD.COM. Otherwise, watchful waiting is recommended until symptoms manifest. AVR is deemed reasonable (Class IIa) in select asymptomatic patients who have **high-risk features**, such as:

- **Very severe AS:** e.g. peak aortic jet velocity ≥5.0 m/s or mean gradient ≥60 mmHg PMC.NCBI.NLM.NIH.GOV.
- **Rapid hemodynamic progression:** e.g. jet velocity increase ≥0.3 m/s per year PMC.NCBI.NLM.NIH.GOV.
- **Elevated biomarkers:** markedly high B-type natriuretic peptide (BNP) levels (≥3× normal) on serial measurements PMC.NCBI.NLM.NIH.GOV.
- **Abnormal exercise test:** development of symptoms, hypotension, or arrhythmia during exercise, indicating unmasked symptoms TCTMD.COM.

Patients without these risk factors are generally managed with close surveillance (clinical and echocardiographic follow-up every 6–12 months).

ESC/EACTS (European) Guidelines (2021): European recommendations align with the above triggers but take a slightly more proactive stance in certain areas. Like ACC, they give a Class I recommendation for AVR in asymptomatic patients who develop LV dysfunction (though ESC uses LVEF ≤50% for Class I)

CARDIOLOGYNEWS.GR. Notably, the ESC/EACTS guidelines consider a **mild reduction in EF as significant** – they add that if LVEF falls below 55% in an asymptomatic patient (even if still above 50%), AVR "should be considered" (Class IIa) CARDIOLOGYNEWS.GR ESCARDIO.ORG. European guidelines also emphasize early surgery for asymptomatic patients with **"very severe" AS or high-risk features** similar to the ACC (very high velocity/gradient, positive exercise test, high BNP, etc.) PMC.NCBI.NLM.NIH.GOV. Both U.S. and European guidelines encourage exercise testing in apparently asymptomatic patients to uncover latent symptoms or drop in blood pressure TCTMD.COM. Overall, current guidelines stop short of recommending routine AVR for *all* asymptomatic severe AS, instead reserving intervention for those with early signs of trouble (declining EF or other risk markers).

Event Risk if Watching: It is important to note that asymptomatic does not mean benign – natural history data show that many "asymptomatic" severe AS patients will declare themselves within 1–2 years. The rate of becoming symptomatic or having adverse events is substantial (approximately 50% or more by 2 years in some series)

CRF.ORG. In the **EARLY-TAVR trial** (discussed below), over 70% of initially asymptomatic patients in the surveillance group required AVR within 2 years as they developed symptoms, often with acute decompensation (e.g. heart failure, syncope) CRF.ORG. Moreover, there is a low but non-zero risk of **sudden cardiac death** during watchful waiting. For instance, in one study of very severe asymptomatic AS, sudden death occurred in ~4% of patients at 4 years and 14% by 8 years without surgery PUBMED.NCBI.NLM.NIH.GOV. These considerations have motivated research into whether *preemptive* AVR could improve survival or quality of life.

Evidence from Recent Randomized Trials

Several key randomized controlled trials (RCTs) in the past few years have compared an early intervention strategy (AVR while asymptomatic) against conservative management in severe AS:

- **RECOVERY Trial (Kang et al., NEJM 2020):** This Korean multicenter RCT enrolled 145 patients with *very severe* asymptomatic AS (AVA ≤0.75 cm^2 and peak velocity ≥4.5 m/s or mean gradient ≥50 mmHg). Patients were randomized to early surgical AVR versus guideline-directed watchful waiting PUBMED.NCBI.NLM.NIH.GOV PUBMED.NCBI.NLM.NIH.GOV. Early surgery led to a dramatic reduction in death from cardiovascular causes. Over a median 4+ year follow-up, the primary outcome (operative or cardiovascular death) occurred in only **1%** of the early-surgery group vs **15%** of the conservative group (HR 0.09, $p<0.01$) PUBMED.NCBI.NLM.NIH.GOV. All-cause mortality was also lower with early AVR (7% vs 21%, HR 0.33) PUBMED.NCBI.NLM.NIH.GOV. Notably, operative mortality in the early AVR group was 0% in this trial PUBMED.NCBI.NLM.NIH.GOV. This was one of the first RCTs to strongly suggest a survival benefit to intervening before symptoms in severe AS.

- **AVATAR Trial (Banovic et al., Circulation 2022):** An international European RCT (157 patients) that included asymptomatic severe AS patients (AVA ≤1.0 cm^2, V_max >4 m/s or mean gradient ≥40) with normal EF. All patients had a **negative exercise stress test** to ensure truly asymptomatic status AHAJOURNALS.ORG AHAJOURNALS.ORG. They were randomized to early surgery (SAVR) vs watchful waiting. After a median ~32 months, the composite primary endpoint (all-cause death, myocardial infarction, stroke, or HF hospitalization) occurred significantly less in the early AVR arm (15% vs 33% of patients; HR 0.46, p=0.02) AHAJOURNALS.ORG AHAJOURNALS.ORG. This 54% relative risk reduction was driven mainly by fewer deaths and heart failure events. There was no statistically significant difference in isolated all-cause mortality between groups (though numerically fewer in the early AVR arm) AHAJOURNALS.ORG. Surgical risk was low: 92% of patients assigned to surgery underwent AVR, with an operative mortality of only 1.4% AHAJOURNALS.ORG. The AVATAR trial provides evidence that early **elective SAVR** can improve outcomes in asymptomatic severe AS, reinforcing the findings of RECOVERY AHAJOURNALS.ORG.

- **EARLY TAVR Trial (Généreux et al., NEJM 2024):** This large North American trial (n=901) extended the question to **transcatheter AVR** in asymptomatic severe AS. Patients (mean age 76, mostly low surgical risk) were randomized to early TAVR vs clinical surveillance CRF.ORG CRF.ORG. Most had truly asymptomatic status confirmed by treadmill testing (90% had negative stress tests) CRF.ORG. At **2 years** (minimum follow-up; median ~3.5 years), early TAVR was superior to watchful waiting. The primary composite endpoint – **death, stroke, or unplanned cardiovascular hospitalization** – occurred in **35.1%** of the TAVR group versus **51.2%** of the surveillance group (HR ~0.50, p<0.001) CRF.ORG. This ~50% risk reduction was driven mainly by fewer **hospitalizations** for heart failure or other cardiac issues in the TAVR arm TCTMD.COM TCTMD.COM. Importantly, early intervention did *not* cause an increase in disabling stroke or death – by 2 years, rates of death or disabling stroke were similar between groups CRF.ORG. Quality-of-life was better with early TAVR: 86.6% of early TAVR patients maintained **favorable health status** (per KCCQ score) vs 68.0% with conservative management (absolute difference ~18%, p<0.001) CRF.ORG. Measures of cardiac function also favored early TAVR, with less progression of LV and left atrial dilation/remodeling at 2 years CRF.ORG. By contrast, in the surveillance group >70% eventually required AVR (mostly after developing symptoms), and ~40% presented with advanced symptoms (NYHA III–IV heart failure, pulmonary edema, syncope) by the time of intervention CRF.ORG. The EARLY TAVR trial thus suggests that in modern practice, preemptive TAVR in asymptomatic patients (particularly older adults at low surgical risk) can prevent clinical deterioration and improve early outcomes CRF.ORG.

- **EVOLVED Trial (Loganath et al., JAMA 2025):** This U.K.-led RCT took a slightly different approach by *selecting asymptomatic patients at high risk of LV decompensation*. EVOLVED enrolled 224 patients with asymptomatic severe AS who showed evidence of myocardial fibrosis on cardiac MRI (mid-wall late gadolinium enhancement) – an indicator of early LV damage TCTMD.COM TCTMD.COM. Participants were randomized to early AVR (SAVR or TAVR, determined by a Heart Team) versus continued watchful management. After a median ~2.5 years, **EVOLVED did *not* find a significant difference** in its primary endpoint (a composite of all-cause death or acute AS-related hospitalization) PUBMED.NCBI.NLM.NIH.GOV PUBMED.NCBI.NLM.NIH.GOV. The primary endpoint occurred in 18% with early intervention vs 23% with conservative care (HR 0.79, p=0.44) PUBMED.NCBI.NLM.NIH.GOV – a trend favoring early AVR but not statistically significant. All-cause mortality was virtually identical (≈14% in both arms over this timeframe) PUBMED.NCBI.NLM.NIH.GOV. However, early AVR did show **benefits in secondary measures**: it significantly reduced *unplanned heart failure hospitalizations due to AS* (6% vs

17%; HR 0.37) PUBMED.NCBI.NLM.NIH.GOV . Additionally, patients who underwent early AVR were less likely to develop symptoms – at 1 year, only 20% of the early-intervention group had any NYHA class II–IV symptoms vs 38% of the conservative group (OR 0.37) PUBMED.NCBI.NLM.NIH.GOV . In other words, early AVR kept patients feeling well longer, but in this trial it did not yet translate to a clear mortality advantage. EVOLVED was somewhat underpowered (it enrolled 224 of a planned 356 patients) and the confidence interval for the primary outcome was wide, leaving open the possibility of benefit not detected PUBMED.NCBI.NLM.NIH.GOV . The authors concluded that using CMR-detected fibrosis alone to guide early AVR did not significantly improve survival or avoid major events in asymptomatic AS PUBMED.NCBI.NLM.NIH.GOV . This more neutral result has added nuance to the debate.

Summary of RCT Findings: Taken together, recent trials generally support a trend toward **earlier intervention** in high-risk asymptomatic severe AS: early AVR consistently *prevented symptom progression and heart failure events*, and in some studies (RECOVERY, AVATAR) significantly improved survival. Not all trials showed a mortality benefit, however. The largest combined data (see meta-analyses below) suggest that early intervention's clearest advantages are in reducing morbid events (hospitalizations, symptom onset), while impacts on long-term mortality are less certain when considering all comers.

Systematic Reviews and Meta-Analyses

To better synthesize the evidence, researchers have performed meta-analyses pooling data from trials and observational studies:

- **Tsampasian et al. (Open Heart/BMJ 2022):** This systematic review/meta-analysis included the two available RCTs at the time (RECOVERY and AVATAR) as well as 8 observational studies of asymptomatic severe AS management. The **pooled RCT data (n=302 patients)** showed that early AVR yielded a **55% relative reduction in all-cause mortality** compared to watchful waiting (HR 0.45, 95% CI 0.24–0.86) PMC.NCBI.NLM.NIH.GOV . Early intervention also reduced the risk of heart-failure hospitalization by 79% (HR 0.21) PMC.NCBI.NLM.NIH.GOV . There was no statistically significant difference in cardiovascular-specific mortality in the RCT subset (perhaps due to few CV deaths overall) PMC.NCBI.NLM.NIH.GOV . The authors noted that the observational studies (over 2,000 patients) likewise favored early AVR, showing improved survival with early treatment

PMC.NCBI.NLM.NIH.GOV. Their conclusion: even in asymptomatic patients, **earlier intervention improves survival and reduces HF events** PMC.NCBI.NLM.NIH.GOV, though they cautioned that further RCT evidence was needed to solidify recommendations PMC.NCBI.NLM.NIH.GOV.

- **Généreux et al. (JACC 2024):** In light of the new trials (EARLY TAVR and EVOLVED), a comprehensive meta-analysis of **4 RCTs** was performed, including ~1,500 patients from RECOVERY, AVATAR, EARLY TAVR, and EVOLVED. This meta, presented in late 2024, found that overall **early AVR did not significantly reduce all-cause or cardiovascular mortality** compared to conservative management when pooling all trials PUBMED.NCBI.NLM.NIH.GOV. However, early AVR was associated with **meaningful reductions in non-fatal events**, particularly unplanned cardiovascular or heart failure hospitalizations and stroke PUBMED.NCBI.NLM.NIH.GOV. In other words, when all RCTs are considered together, the mortality benefits appear neutral on average (likely influenced by the larger neutral EVOLVED trial and the relatively short follow-ups), but patients clearly have fewer complications and hospital admissions if the valve is addressed before symptoms. These results underscore that the benefit of early intervention may lie in improved quality of life and avoidance of acute decompensation, more so than in immediate survival advantage TCTMD.COM TCTMD.COM.

It’s worth noting that all analyses agree early AVR is not causing *harm* in terms of early mortality – modern SAVR and TAVR can be performed with low periprocedural risk in asymptomatic patients. The debate centers on *degree of benefit* and patient selection.

Key Outcomes: Early AVR vs Watchful Waiting

- **Mortality:** Early surgical AVR has shown a mortality benefit in some studies (e.g. a 68% reduction in CV death in RECOVERY PUBMED.NCBI.NLM.NIH.GOV). Pooled data are mixed – earlier meta-analyses indicated improved survival PMC.NCBI.NLM.NIH.GOV, while the latest evidence with more patients shows no statistically significant difference in mortality overall PUBMED.NCBI.NLM.NIH.GOV. Importantly, none of the RCTs showed an *excess* mortality with early intervention. At worst, early AVR confers equal survival to waiting (as in EVOLVED) PUBMED.NCBI.NLM.NIH.GOV, and at best it significantly prolongs life (as in Kang et al.) PUBMED.NCBI.NLM.NIH.GOV. Many experts believe that with longer follow-up, early AVR will translate to a survival gain, given it prevents late

complications; however, proving a mortality benefit requires large sample sizes and time.

- **Sudden Death Prevention:** One rationale for early AVR is preventing sudden cardiac death during the asymptomatic phase. Sudden death in asymptomatic severe AS is relatively infrequent but does occur. In conservative arms of studies, a small percentage of patients died suddenly while waiting (e.g. in RECOVERY, several sudden deaths occurred in year 5–8 of follow-up PUBMED.NCBI.NLM.NIH.GOV). Early AVR virtually eliminates this risk by treating the mechanical obstruction before such an event can happen. Thus, for a patient with severe AS who otherwise feels well, the **trade-off** is between a low but real risk of catastrophic sudden death on watchful waiting versus the upfront procedural risk of AVR.

- **Symptom Onset and Progression:** Consistently, early intervention delays or averts the development of symptoms. In the trials, patients who did not get early AVR almost inevitably became symptomatic and required valve replacement within a couple of years CRF.ORG. For example, in EARLY TAVR, nearly half of the surveillance group progressed to symptom-triggered AVR by 1 year, and >70% by 2 years CRF.ORG. EVOLVED showed that one year after randomization, significantly more patients in the conservative group had developed NYHA class II+ symptoms compared to those who had early AVR (38% vs 20%) PUBMED.NCBI.NLM.NIH.GOV. Early AVR thus **preserves asymptomatic status longer** – essentially, it trades an asymptomatic surgery now to prevent a symptomatic decline later.

- **Quality of Life (QoL):** Asymptomatic patients have high baseline QoL, but those who undergo early AVR tend to maintain excellent QoL, whereas those under watchful waiting can experience a decline once symptoms emerge. In EARLY TAVR, patient-reported health status (Kansas City Cardiomyopathy Questionnaire) was significantly better in the early TAVR arm through follow-up CRF.ORG. A larger proportion of early TAVR patients reported no limitation and high quality-of-life scores. Moreover, early AVR can **prevent the period of poor health** that many in the conservative group experience when they present with advanced symptoms (e.g. acute heart failure) prior to eventual rescue AVR CRF.ORG. By intervening at a planned stage, patients avoid the morbidity of decompensation. Overall, data indicate early AVR *improves or maintains quality of life* in asymptomatic AS compared to delaying until symptoms occur TCTMD.COM TCTMD.COM.

- **LV Function and Long-Term Cardiac Health:** Chronic pressure overload in AS leads to LV hypertrophy, fibrosis, and eventual dysfunction. One argument for early AVR is to **prevent irreversible myocardial damage**. Subclinical LV strain impairment and fibrosis can be present even when EF is normal PMC.NCBI.NLM.NIH.GOV PMC.NCBI.NLM.NIH.GOV. If AVR is done early, it may allow more complete recovery of LV structure and function. Trials have measured indices of cardiac remodeling – for instance, EARLY TAVR reported better preservation of left ventricular ejection fraction and less increase in left atrial size by 2 years with early intervention CRF.ORG. The concept of "staging" AS by cardiac damage (fibrosis, diastolic dysfunction, pulmonary pressure, etc.) is gaining traction. Early AVR before advanced damage occurs could improve long-term outcomes (even if immediate mortality is unchanged) by reducing progression to heart failure. This hypothesis is supported by biomarker and imaging studies showing that fibrosis accumulates rapidly once it starts and is **irreversible despite later AVR** PMC.NCBI.NLM.NIH.GOV PMC.NCBI.NLM.NIH.GOV. Thus, intervening before extensive fibrosis (the premise of EVOLVED) could theoretically improve long-term heart failure–free survival, although EVOLVED's neutral result suggests fibrosis alone may not be a sufficient trigger or that more patients are needed to confirm this approach.

- **Procedural Risk:** In the past, surgery in an asymptomatic patient was a hard sell because AVR itself carries risk. But surgical techniques and patient selection have improved such that **operative mortality for isolated AVR is often 1–2% at experienced centers**, and even lower in the recent trials (0–1.4% in RECOVERY/AVATAR) PUBMED.NCBI.NLM.NIH.GOV AHAJOURNALS.ORG. TAVR, being less invasive, has further reduced the threshold for intervention – in EARLY TAVR, there was no increase in stroke or death with the upfront TAVR strategy CRF.ORG. The main risks of TAVR (like need for pacemaker or vascular complications) were not highlighted as prohibitive in recent studies. Therefore, the **safety profile of early AVR is favorable**, making it reasonable to consider in an asymptomatic patient who is otherwise a good candidate. The decision hinges on weighing this low procedure risk against the aforementioned risks of waiting. Patient age and comorbidities are factors: an elderly patient might opt for a low-risk TAVR now rather than gamble on deterioration, whereas a younger patient might be monitored a bit longer if surgical AVR (with its prosthetic valve longevity considerations) is the only option.

Areas of Conflicting Evidence and Ongoing Debate

Despite the accumulation of positive trials, there remains some disagreement in the cardiology community and variability in recommendations:

- **Guideline Evolution:** Current guidelines are cautious – they stop short of endorsing routine early AVR for all asymptomatic severe AS, instead recommending it only in high-risk subsets (EF <50%, abnormal exercise test, very high gradients, etc.) TCTMD.COM PMC.NCBI.NLM.NIH.GOV. However, the new evidence has begun to shift opinion. The European guidelines have already expanded certain indications (e.g., considering surgery at EF <55% CARDIOLOGYNEWS.GR), reflecting a **trend toward earlier intervention**. U.S. guidelines (ACC/AHA 2020) were published just as the first RCT evidence emerged, so they remain more conservative, listing some scenarios as Class IIa but generally favoring watchful waiting in truly asymptomatic, normal-EF patients TCTMD.COM. We may see guideline updates in the near future incorporating trials like AVATAR, EARLY TAVR, and EVOLVED. If the weight of evidence continues to favor early AVR, future guidelines could move those indications from "reasonable" (Class IIa) to a stronger recommendation for selected patients, though a blanket Class I for all asymptomatic severe AS is unlikely without clear mortality benefit and cost-effectiveness data.

- **Patient Selection – Not All Asymptomatic AS Is Equal:** A key area of debate is how to identify which "asymptomatic" patients will benefit most from early AVR. Severe AS encompasses a spectrum – some patients have extremely high gradients and subclinical symptoms, essentially "symptomatic-in-waiting," while others may truly feel fine and have less extreme valve measurements. Trials like RECOVERY focused on **very severe** AS, where the event risk is highest PUBMED.NCBI.NLM.NIH.GOV PUBMED.NCBI.NLM.NIH.GOV. It's generally agreed that those patients (V_max >5 m/s, etc.) have the most to gain from early AVR, and guidelines already lean in that direction (Class IIa) PMC.NCBI.NLM.NIH.GOV. On the other hand, a patient with severe AS at the threshold (say AVA 0.9 cm^2, gradient ~40) who is completely asymptomatic and has none of the high-risk markers might reasonably be observed, since their short-term risk is lower. The **EVOLVED trial's neutral outcome** highlights that even among high-risk features (fibrosis in that case), if patients are a bit older with only moderate gradients (mean V_max was 4.3 m/s) PUBMED.NCBI.NLM.NIH.GOV PUBMED.NCBI.NLM.NIH.GOV, immediate AVR may not improve hard outcomes in the short term. This suggests we need a nuanced, individualized approach – not all asymptomatic severe AS should be lumped together.

- **Conflicting Trial Results:** As detailed, RECOVERY and AVATAR showed significant outcome benefits with early SAVR PUBMED.NCBI.NLM.NIH.GOV AHAJOURNALS.ORG, whereas EVOLVED did not show a primary endpoint benefit (though it did show fewer hospitalizations and symptoms) PUBMED.NCBI.NLM.NIH.GOV PUBMED.NCBI.NLM.NIH.GOV. EARLY TAVR showed clear benefits in reducing events but primarily driven by hospitalizations, not a mortality drop at 3 years TCTMD.COM TCTMD.COM. How does one reconcile these differences? Patient populations and criteria differed: RECOVERY/AVATAR patients were slightly younger (mid-60s) with very high gradients, likely poised to benefit from surgery before something bad happened. EVOLVED patients were older (mean 73) with evidence of fibrosis but only moderate "severe" gradients (~45 mmHg mean), possibly representing a later stage of the disease where some damage is already done but competing risks of age are higher. As one commentator noted, "it's hard to take someone who's doing well and make them better" if their risk profile is intermediate and the trial is small TCTMD.COM TCTMD.COM. The **duration of follow-up** also matters: benefits of early AVR might manifest over longer periods than some trials observed. The conflict in evidence underscores that more data (and longer follow-up) will help determine if early AVR unequivocally improves survival, or if its main advantage is avoiding symptoms and hospital stays.

- **Role of TAVR vs SAVR:** With TAVR becoming widely available, some argue that an asymptomatic patient who might not have opted for early **surgery** could be a good candidate for early **TAVR** (since it's less invasive). The decision between SAVR and TAVR in asymptomatic AS is influenced by age and valve anatomy. Guidelines currently favor TAVR in older patients (e.g. >75–80 years) and SAVR in younger patients due to prosthetic valve durability concerns CARDIOLOGYNEWS.GR CARDIOLOGYNEWS.GR. The trials reflect this: EARLY TAVR's average patient was 76, whereas RECOVERY's was ~64. Thus, in practice, an older asymptomatic patient may be more likely to undergo early TAVR if intervention is chosen, whereas a 60-year-old might undergo SAVR or continued observation. The good news is that both modalities have been shown to be safe in asymptomatic patients. Ongoing studies (and real-world data) will further clarify long-term **valve durability** and any procedure-specific pros/cons in asymptomatic cohorts. For instance, a consideration is that performing TAVR very early starts the clock on a tissue valve that might need re-intervention in a couple of decades if the patient is relatively young. These nuances factor into decision-making.

- **Future Directions – Biomarkers and Advanced Imaging:** Research is ongoing to improve risk stratification of asymptomatic AS. Aside from EF, BNP, and valve hemodynamics, things like **global longitudinal strain** (GLS on echo) and **cardiac MRI fibrosis** are being studied as triggers for AVR. EVOLVED tested the MRI fibrosis hypothesis and did not find a major outcome difference PUBMED.NCBI.NLM.NIH.GOV, suggesting that using fibrosis alone may not yet justify intervention. Other trials (e.g., the ongoing DANAVR study mentioned in some literature PMC.NCBI.NLM.NIH.GOV) are combining multiple markers (echo measures of LA size, GLS, BNP, etc.) to select patients for early surgery. There is also interest in **genomic and molecular markers** that might identify whose stenosis is likely to progress fastest. Until these tools mature, clinicians rely on the established clinical and echocardiographic indicators and careful patient assessment (including exercise testing) to guide management.

Conclusion

Balanced Perspective: The question of routine early intervention in asymptomatic severe AS is complex. On one hand, **compelling evidence** now shows that many patients benefit from earlier AVR – they are less likely to suffer sudden death, less likely to develop heart failure, and more likely to maintain a good quality of life PUBMED.NCBI.NLM.NIH.GOV CRF.ORG. Early AVR can be done with low risk and prevents the period of morbidity associated with waiting for symptoms CRF.ORG PUBMED.NCBI.NLM.NIH.GOV. On the other hand, **not every asymptomatic patient is high-risk**, and some can be safely monitored for a time. There are legitimate reasons to avoid premature intervention in certain cases, such as the absence of any high-risk features, very advanced age or comorbidities that make even a low-risk procedure less worthwhile, or patient preference to defer surgery absent symptoms. The evidence is still evolving: while early AVR clearly reduces **morbidity**, the overall **mortality benefit** remains an area of active investigation and debate PUBMED.NCBI.NLM.NIH.GOV.

In practice, current guidelines and experts take a **selective approach** – identify asymptomatic patients who have indicators of imminent trouble (falling EF, extreme valve gradient, positive exercise test, etc.) and intervene in those, rather than blanket treat all asymptomatic AS. This “watchful waiting with intent to intervene at the earliest sign of risk” strategy is supported by both past practice and the latest trials (which largely enrolled such higher-risk asymptomatic patients). However, with TAVR expanding and surgical outcomes improving, the threshold to intervene is lowering. The most recent and high-impact studies tilt the scale toward earlier intervention in many patients, and we are likely witnessing a shift in the standard of care. For now, a **patient-tailored decision** is crucial: a Heart Team should weigh the patient’s surgical/TAVR risk, age, valve anatomy, and any risk markers (EF, BNP, gradient, etc.), and discuss with the patient the trade-offs between an elective procedure now vs. observation. In asymptomatic severe AS, **“doing nothing” is not truly benign** – close follow-up is required, and an individualized plan should be in place for prompt AVR at the appropriate time. The accumulated evidence suggests that *for most patients with truly severe AS, it is safer to replace the valve a bit too early than a bit too late*. Future guideline updates will likely reflect this emerging consensus as more data continue to affirm the long-term benefits of early intervention in the appropriate asymptomatic patient population.

Sources:

- Kang DH *et al.* (RECOVERY Trial), *N Engl J Med* 2020 – Early SAVR vs. conservative in asymptomatic very severe AS PUBMED.NCBI.NLM.NIH.GOV PUBMED.NCBI.NLM.NIH.GOV
- Banovic M *et al.* (AVATAR Trial), *Circulation* 2022 – Early SAVR vs. conservative in asymptomatic severe AS AHAJOURNALS.ORG AHAJOURNALS.ORG
- Généreux P *et al.* (EARLY TAVR Trial), TCT 2024/ *NEJM* 2024 – Early TAVR vs. surveillance in asymptomatic severe AS CRF.ORG CRF.ORG
- Loganath K *et al.* (EVOLVED Trial), *JAMA* 2025 – Early AVR vs. conservative in asymptomatic severe AS with fibrosis PUBMED.NCBI.NLM.NIH.GOV PUBMED.NCBI.NLM.NIH.GOV
- 2020 ACC/AHA Valvular Heart Disease Guideline – Recommendations for asymptomatic severe AS management TCTMD.COM
- 2021 ESC/EACTS Valvular Guidelines – Recommendations for asymptomatic severe AS (earlier intervention triggers) CARDIOLOGYNEWS.GR ESCARDIO.ORG
- Tsampasian V *et al.*, *Open Heart* 2022 – Meta-analysis of early intervention vs. watchful waiting PMC.NCBI.NLM.NIH.GOV

- Généreux P *et al.*, *J Am Coll Cardiol* 2024 – Meta-analysis of 4 RCTs (early AVR vs. surveillance) PUBMED.NCBI.NLM.NIH.GOV
- TCTMD and AHA news summaries – Context and expert commentary on EARLY TAVR & EVOLVED trials TCTMD.COM TCTMD.COM .

Aortic Stenosis Intervention Evidence

I am trying to determine whether there is sufficient evidence to support routine intervention (either by transcatheter aortic valve replacement or surgical aortic valve replacement) for aortic stenosis that is severe but asymptomatic.

Are you looking for evidence from clinical trials, observational studies, guidelines from medical societies, or a combination of these sources? Additionally, do you have a preferred timeframe for the research (e.g., recent studies from the last 5–10 years)? Let me know if you'd like a focus on specific patient populations, such as elderly patients or those with particular comorbidities.

Both primary research, guidelines, systematic reviews and meta-analyses. When there are conflicting positions I want these highlighted. I have no preference with respect to the source of the information geographically. Limiting the research to the last 5-10 years is reasonable.

I will conduct a comprehensive review of the latest evidence (from the last 5–10 years) regarding routine intervention for severe but asymptomatic aortic stenosis. This will include primary research, clinical guidelines, systematic reviews, and meta-analyses. I will also highlight any conflicting positions within the literature.

I will get back to you once I have gathered and synthesized the relevant information.

Background

Severe aortic stenosis (AS) can remain clinically “asymptomatic” even when the valve obstruction is advanced. Traditionally, management of asymptomatic severe AS has been conservative (“watchful waiting”) until symptom onset, given the procedural risks of surgical aortic valve replacement (SAVR). This approach was supported by earlier data suggesting relatively low (~1% per year) sudden death rates in truly asymptomatic patients and a belief that early surgery might confer little survival benefit if patients feel well. However, over the past 5–10 years, several developments have challenged this paradigm: operative risks have declined (especially with less invasive transcatheter AVR, TAVR), and emerging evidence indicates subclinical cardiac damage may progress even before overt symptoms. The key question has become whether **routine early intervention (SAVR or TAVR)** in severe asymptomatic AS improves outcomes compared to waiting for symptoms. Below is a comprehensive review of recent evidence (clinical trials, meta-analyses) and current guideline recommendations, highlighting consensus and controversy in the field.

Key Randomized Trials (2018–2024)

Recent RCTs have directly compared early intervention to conservative management in asymptomatic severe AS – a major advance in evidence for this long-debated scenario:

- **RECOVERY Trial (NEJM 2020, Kang et al.)** – This Korean multicenter trial enrolled 145 patients with *very severe* asymptomatic AS (aortic valve area $\leq$0.75 cm^2 and jet velocity $\geq$4.5 m/s or mean gradient $\geq$50 mmHg). Patients were randomized to early SAVR versus guideline-directed conservative care. Early surgery yielded a striking reduction in the primary endpoint (operative or CV death): **1% vs 15%** event rate at ~6 years (HR 0.09, p = 0.003) PUBMED.NCBI.NLM.NIH.GOV. All-cause mortality was also lower with early SAVR (7% vs 21%, HR 0.33) PUBMED.NCBI.NLM.NIH.GOV. Notably, 95% of patients assigned to early surgery underwent AVR (none died perioperatively) PUBMED.NCBI.NLM.NIH.GOV. This landmark trial provided the first RCT evidence that **preemptive SAVR in very severe AS can significantly improve survival** compared to waiting for symptoms PUBMED.NCBI.NLM.NIH.GOV. It is limited by a highly selected population (very severe AS, relatively young cohort) but strongly supports early intervention in that subgroup.

- **AVATAR Trial (Circulation 2022, Banovic et al.)** – An international European RCT (9 centers) that randomized 157 patients with asymptomatic *severe* AS (AVA ≤1.0 cm², velocity >4.0 m/s, mean gradient ≥40 mmHg; normal LVEF; all had a negative exercise test to confirm truly asymptomatic status) to early SAVR vs conservative management PUBMED.NCBI.NLM.NIH.GOV PUBMED.NCBI.NLM.NIH.GOV. Median follow-up was ~32 months PUBMED.NCBI.NLM.NIH.GOV. The **primary composite outcome** (all-cause death, acute MI, stroke, or unplanned heart failure hospitalization) occurred significantly less in the early surgery group (13 events) than in the conservative group (26 events), **HR 0.46, *p* = 0.02** PUBMED.NCBI.NLM.NIH.GOV PUBMED.NCBI.NLM.NIH.GOV. In intention-to-treat analysis, early SAVR reduced the composite risk by ~54%. There was **no statistically significant difference in all-cause mortality alone** at this follow-up, and other secondary endpoints (first HF hospitalization, major bleeding, etc.) did not reach significance, though all trended favorably for early AVR PUBMED.NCBI.NLM.NIH.GOV. Operative mortality for SAVR was low (1.4%) PUBMED.NCBI.NLM.NIH.GOV. **Conclusion:** Early SAVR in asymptomatic severe AS reduced major adverse events (driven largely by prevention of heart failure and urgent surgery) PUBMED.NCBI.NLM.NIH.GOV PUBMED.NCBI.NLM.NIH.GOV. While a mortality benefit was not definitively shown in this moderate-sized trial, AVATAR reinforced that early surgery is safe and can improve outcomes beyond symptom relief.

- **EARLY TAVR Trial (NEJM/TCT 2024, Généreux et al.)** – The first large RCT of *transcatheter* AVR in asymptomatic AS. This North American trial randomized **901 patients** (mean age 76, mostly low surgical risk) with asymptomatic severe AS (EF ≥50%, rigorously confirmed truly asymptomatic by treadmill testing) to early TAVR vs clinical surveillance CRF.ORG CRF.ORG. At a median follow-up of 3.8 years, the **primary endpoint** – a composite of *death, stroke, or unplanned cardiovascular hospitalization* – occurred in 35.1% of the early TAVR group versus 51.2% with initial surveillance (**HR ~0.50, *p*<0.001**) CRF.ORG. This 50% relative risk reduction was driven mainly by **fewer unplanned hospitalizations** in the early TAVR arm TCTMD.COM. In fact, >70% of patients in the surveillance group wound up undergoing AVR within 2 years as they developed symptoms, often emergently (median time to crossing over was ~11 months) TCTMD.COM CRF.ORG. Importantly, **no significant difference in mortality or disabling stroke** emerged between groups over this follow-up – early intervention incurred no penalty in these hard outcomes CRF.ORG. Early TAVR patients had better maintained LV and left atrial function and higher likelihood of preserved quality-of-life (KCCQ score) CRF.ORG CRF.ORG. **Key takeaway:** In older low-risk patients, an early TAVR strategy was safe and halved the rate of the composite endpoint by **preventing symptomatic deterioration and urgent interventions**,

although *overall survival was similar* at ~4 years CRF.ORG. This trial provides strong evidence that proactive TAVR can forestall heart failure and other complications in asymptomatic severe AS.

- **EVOLVED Trial (JAMA 2024, Loganath et al.)** – This UK-led RCT took a **biomarker-guided approach**: 225 patients with asymptomatic severe AS were screened for subclinical LV dysfunction (by elevated high-sensitivity troponin or ECG strain) and underwent cardiac MRI; those with evidence of mid-wall fibrosis (late gadolinium enhancement) were randomized to early AVR (SAVR or TAVR per Heart Team) vs standard watchful waiting TCTMD.COM TCTMD.COM. The hypothesis was that patients showing fibrosis – an indicator of early myocardial damage – might especially benefit from early valve replacement. After a median ~2 years, **early intervention did *not* significantly reduce the primary outcome** (all-cause death or unplanned AS-related hospitalization) compared to conservative management (HR 0.79, 95% CI 0.44–1.43, $p = 0.43$) TCTMD.COM. All-cause mortality was similar in both arms TCTMD.COM. However, early AVR did yield a 63% relative reduction in *unplanned AS-related hospitalizations* (HR 0.37) TCTMD.COM – consistent with other trials showing fewer urgent HF admissions or emergent valve surgeries when intervening early. Notably, even in the surveillance group, 77% of patients ultimately underwent AVR during follow-up (typically once symptoms developed) TCTMD.COM. **Implications:** In this high-risk subset (asymptomatic but with fibrosis), early AVR did not improve short-term survival, suggesting that the presence of myocardial fibrosis alone may not be enough to justify intervention in the absence of symptoms TCTMD.COM. The reduction in hospitalizations echoes the benefit of averting acute decompensation, but EVOLVED is a reminder that identifying exactly *who* benefits most from early intervention remains challenging.

Summary of RCT Evidence: Taken together, these trials indicate that in *selected* asymptomatic severe AS patients, **early AVR (whether SAVR or TAVR) is feasible and substantially reduces the risk of heart failure, syncope, and urgent hospitalizations/surgeries** compared to waiting. The trade-off is that an upfront procedure (with its small inherent risks) is done on a patient who currently feels well. So far, absolute **mortality benefits have been inconsistent**: one trial in an extreme subset (very severe AS) showed a clear survival gain PUBMED.NCBI.NLM.NIH.GOV, whereas others (with broader inclusion criteria) have shown *no significant difference in all-cause mortality* over ~3–4 years PUBMED.NCBI.NLM.NIH.GOV TCTMD.COM. Importantly, none

of the trials show a mortality *penalty* for early intervention – procedure-related risks in modern practice are low, and early AVR has not increased stroke, MI, or death compared to conservative management PUBMED.NCBI.NLM.NIH.GOV PUBMED.NCBI.NLM.NIH.GOV. The benefit of early intervention is most apparent in preventing patients from deteriorating into frank symptoms or heart failure (which can occur sooner than expected – in EARLY TAVR, ~50% became symptomatic within 1 year of observation) TCTMD.COM. The question of improved long-term survival with early AVR may require longer follow-up or larger studies, but the available RCT data have already influenced guidelines toward a more proactive stance (especially given the **"no harm"** and quality-of-life improvements seen with early treatment).

Systematic Reviews & Meta-Analyses

To synthesize the growing evidence, several meta-analyses in recent years have compared **early AVR vs conservative management** in asymptomatic severe AS:

- **Heart 2023 Meta-Analysis (Costa et al.)** – This meta-analysis pooled **12 studies (2 RCTs and 10 observational/cohort studies)** with over 4,100 asymptomatic severe AS patients PUBMED.NCBI.NLM.NIH.GOV. Early AVR (surgery) was compared to watchful waiting. The pooled results favored early intervention: **all-cause mortality was significantly lower** with an early AVR strategy (pooled OR ~0.40, 95% CI 0.35–0.45) than with watchful waiting PUBMED.NCBI.NLM.NIH.GOV. Early AVR also had lower cardiovascular mortality (OR ~0.33) and markedly fewer heart failure hospitalizations (OR ~0.19) PUBMED.NCBI.NLM.NIH.GOV. Notably, **stroke rates were similar** between early and conservative approaches (no statistically significant difference, OR ~1.3, p = 0.36) PUBMED.NCBI.NLM.NIH.GOV, and rates of myocardial infarction were also not significantly different PUBMED.NCBI.NLM.NIH.GOV. These findings suggest that intervening before symptoms can indeed prolong life and reduce cardiac complications, without adding neurologic risk, **when considering the totality of evidence**. However, heterogeneity was high (I^2 ~60% for mortality), reflecting differences between studies PUBMED.NCBI.NLM.NIH.GOV. Much of the mortality benefit signal came from observational data and the RECOVERY trial; in contrast, smaller RCTs alone have less dramatic effects. Overall, this analysis supports **early surgery as associated with better survival and less HF**, but the authors caution that patient selection and study design differences drive some variability.

- **Open Heart/BMJ 2024 Meta-Analysis (Senguttuvan et al.)** – This recent review focused on both RCT and observational evidence, with an emphasis on analyzing the *highest-quality data separately*. In the **RCT-only analysis**, pooling available trials (largely RECOVERY and AVATAR at the time) found **no statistically significant difference in all-cause mortality** between early AVR vs conservative management (incidence rate ratio ~0.50, p = 0.09) PUBMED.NCBI.NLM.NIH.GOV. In other words, when considering strictly randomized data, early intervention *trended* toward fewer deaths but did not meet significance – likely due to limited sample size and follow-up (and the fact that one RCT showed large benefit while another showed none). However, when observational studies were included, the meta-analysis did find a **mortality benefit for early AVR (pooled IRR ~0.4, p<0.01)**, albeit with very high heterogeneity PUBMED.NCBI.NLM.NIH.GOV. Consistent with other findings, early AVR was clearly beneficial in reducing other endpoints: across studies, it **significantly lowered rates of MACE, cardiac death, sudden death, symptom development, and HF hospitalization** compared to initial conservatism PUBMED.NCBI.NLM.NIH.GOV. There was **no difference in MI, stroke, or major bleeding rates** between early vs late intervention groups PUBMED.NCBI.NLM.NIH.GOV. The authors concluded: while **early AVR does not yet show a proven all-cause mortality reduction in RCTs alone**, it **does prevent heart-failure-related events and likely improves CV outcomes overall** PUBMED.NCBI.NLM.NIH.GOV. They highlight the "moderate" quality of evidence for mortality (hence guidelines remain cautious), but point to the consistent reduction in HF admissions and late symptom burden as strong arguments for earlier intervention PUBMED.NCBI.NLM.NIH.GOV PUBMED.NCBI.NLM.NIH.GOV.

- **Other Reviews:** A number of narrative reviews and smaller analyses echo similar themes. For example, an Open Heart 2024 review noted that when strictly examining randomized data, the mortality difference is not definitive, reinforcing that the **"benefit" of early AVR is chiefly in avoiding irreversible cardiac damage and acute decompensation rather than immediately extending life** PUBMED.NCBI.NLM.NIH.GOV. Conversely, large registry analyses (propensity-matched) have suggested survival advantages to early surgery, indicating that in real-world practice carefully selected asymptomatic patients often fare better with prompt AVR PUBMED.NCBI.NLM.NIH.GOV. Overall, the literature is converging on the notion that **waiting for symptoms entails a real risk** – up to 30–50% of "asymptomatic" patients will declare symptoms or require urgent AVR within 1–2 years TCTMD.COM – and that *proactive AVR can mitigate this with minimal added risk.* The meta-analyses underline that, aside from the operative period, **early intervention doesn't increase stroke or MI**, addressing a key

historical concern.

Clinical Guideline Recommendations (ACC/AHA vs ESC)

Major cardiology societies have updated their valve disease guidelines in light of emerging evidence, though full consensus is still evolving. Both the American (ACC/AHA) and European (ESC/EACTS) guidelines in the last few years have inched toward earlier intervention for asymptomatic AS, with nuanced criteria:

- **ACC/AHA (U.S.) Guidelines 2020:** The American guidance (2020 Valve Disease Guideline) continues to stratify asymptomatic severe AS as Stage C (C1 = asymptomatic with normal EF, C2 = asymptomatic with LV dysfunction). It maintains that *symptom onset* or *LV systolic dysfunction* are firm triggers for AVR (Class I). Specifically, **AVR (SAVR or TAVR) is a Class I recommendation for asymptomatic severe AS if LVEF <50%** (even without symptoms) TCTMD.COM, or if the patient is undergoing other cardiac surgery (e.g. CABG) – reflecting clear benefit in those situations. Additionally, **exercise testing is emphasized**: if an asymptomatic patient develops symptoms during exercise or has a drop in blood pressure with exercise, that unmasked symptom counts – AVR is indicated (Class I) PMC.NCBI.NLM.NIH.GOV. Beyond these, the ACC/AHA introduces **"trigger" factors where early AVR is *reasonable*** even before symptoms (Class IIa). These factors include:

  - **Very severe AS** – defined by a high transvalvular velocity (≥5.0 m/s) or mean gradient ≥60 mmHg – in a patient at low surgical risk PMC.NCBI.NLM.NIH.GOV. Early AVR is considered reasonable (IIa) in this scenario given the higher risk of rapid deterioration.
  - **Rapid hemodynamic progression** – e.g. an increase in peak velocity ≥0.3 m/s per year on serial echos (again in a low-risk patient) PMC.NCBI.NLM.NIH.GOV. Rapidly worsening valve obstruction portends imminent symptoms, so proactive surgery is reasonable (IIa).
  - **Elevated biomarkers** – notably, a significantly raised B-type natriuretic peptide (BNP) level (≥3 times normal for age/gender) in an asymptomatic patient, *confirmed on repeat tests*, is a IIa criterion for AVR PMC.NCBI.NLM.NIH.GOV. High BNP indicates ventricular strain and correlates with worse outcomes.

- Some experts also consider extreme LV hypertrophy or pulmonary hypertension at rest as additional factors (ESC explicitly lists pulmonary systolic pressure >60 mmHg as a consideration, whereas ACC implies it in the context of “stage C” features).

In summary, ACC/AHA 2020 urges **close monitoring** of asymptomatic severe AS and states that if any high-risk features are present, **early AVR is “reasonable” (Class IIa)** TCTMD.COM . Otherwise, continued surveillance (every 6–12 months clinical/echo follow-up) is advised for truly asymptomatic, stable patients. The guidelines did not yet give a blanket endorsement for TAVR in asymptomatic patients, but since they say “AVR” one can interpret that the mode (SAVR vs TAVR) should follow the usual risk-based algorithm (younger low-risk patients toward surgery, older high-risk patients toward TAVR – see below). Notably, U.S. recommendations at that time were conservative about intervening *purely* for asymptomatic status, unless these risk markers or a positive exercise test were present TCTMD.COM .

- **ESC/EACTS (European) Guidelines 2021:** The European guidelines likewise shifted toward earlier intervention, with a few differences in thresholds. One semantic change: they replaced the term “surgical valve replacement” with **“intervention”** when discussing asymptomatic AS, to acknowledge that **TAVR may be an appropriate intervention in select asymptomatic patients as evidence evolves** REVESPCARDIOL.ORG . Key recommendations:

  - **LVEF trigger:** ESC made the cutoff slightly less strict – they give a Class I indication for AVR if LVEF *drops* **≤50%** (similar to ACC), and notably they added that AVR *“should be considered” (Class IIa)* even if **LVEF is in the 50–55% range** for asymptomatic patients REVESPCARDIOL.ORG . This reflects concern that mild LV dysfunction is already a sign of decompensation. (By contrast, ACC still uses <50% as the trigger and doesn’t explicitly mention 50–55% range as an indication.)
  - **Exercise testing:** If an asymptomatic patient has *demonstrable symptoms on exercise testing*, the ESC considers that a Class I indication to intervene (Level C evidence) – essentially the patient is not truly asymptomatic PMC.NCBI.NLM.NIH.GOV .
  - **“Very severe” AS:** The ESC defines this more stringently now – they use peak velocity >5.5 m/s as the cutoff for “very severe” (whereas ACC uses ≥5.0). For such very severe AS in a low-risk patient, **early intervention “should be considered” (Class IIa)** given the high likelihood of poor outcome if waiting REVESPCARDIOL.ORG . This

threshold change (>5.5) actually narrowed the criterion slightly, indicating ESC wanted only the most extreme cases in this group (the 2017 ESC guideline had used >5.0 m/s).

- **Valve calcification & progression:** The presence of **severe valve calcification with a rapid increase in gradient** is listed as a sign of risk – an asymptomatic patient meeting these criteria *should be considered* for AVR (Class IIa) REVESPCARDIOL.ORG. Similarly, a significantly elevated BNP is acknowledged as a risk marker warranting consideration of early intervention (IIa). The level of evidence for these risk-factor criteria was upgraded to B (from C) in 2021 due to the supportive but non-definitive data REVESPCARDIOL.ORG.
- Additionally, ESC recommends AVR (Class I) in any asymptomatic severe AS patient who is undergoing other cardiac surgery (valve or CABG), echoing ACC. They also caution that if a patient with severe AS needs noncardiac surgery, that case should be carefully managed – if the noncardiac procedure is elective and the AS poses high risk, **do the AVR first if feasible** REVESPCARDIOL.ORG.

In summary, **ESC 2021 broadened the indications for asymptomatic severe AS modestly**: it kept true "watchful waiting" for those with absolutely no risk markers, but gave Class IIa endorsements to intervene for LVEF deterioration even if mild, very high valve velocity, rapid progression, or high BNP REVESPCARDIOL.ORG. This is largely aligned with ACC's IIa criteria (with minor differences in cutoff values). One notable aspect is ESC's explicit inclusion of the word "intervention" – signalling that as trials like EARLY TAVR report positive results, transcatheter therapy could be directly recommended in future guidelines for asymptomatic patients (the ESC authors anticipated ongoing studies might justify TAVI in asymptomatic AS REVESPCARDIOL.ORG).

- **Other Guidelines/Global:** Other national guidelines generally parallel these positions. For example, the Canadian Cardiovascular Society (CCS) has in recent years also endorsed early surgery for asymptomatic severe AS if there is LVEF <50%, abnormal exercise test, very high gradients, or markedly elevated BNP, and they encourage exercise testing to unmask symptoms. The UK's NICE guideline (last updated prior to these RCTs) has been more conservative, but likely will incorporate new evidence in future updates. Overall, there is growing international consensus in guidelines that **certain asymptomatic patients should be treated without waiting for frank symptoms**. All guidelines stress the importance of a Heart Team approach and shared decision-making, especially as patient values play a role (an informed asymptomatic patient might opt for early TAVR to avoid risk of sudden decompensation, or conversely might prefer to defer intervention until absolutely necessary). Mode of intervention (TAVR vs SAVR) is generally determined by age and surgical risk in line with each guideline's recommendations for symptomatic AS. For instance, ESC 2021 introduced **age cut-offs**: for symptomatic AS, <75 years and low risk favors SAVR, whereas ≥75 or high-risk favors TAVI (Class I for each) TCTMD.COM TCTMD.COM. For asymptomatic patients, currently the decision to intervene is the bigger question; once the decision **to intervene** is made, similar age/risk considerations would apply in choosing TAVR vs SAVR, as long as both options are viable and the patient's anatomy is suitable REVESPCARDIOL.ORG REVESPCARDIOL.ORG.

Areas of Consensus

Based on the evidence and guidelines, several points of **broad agreement** have emerged regarding asymptomatic severe AS:

- **Symptom Onset is Dangerous – Don't Delay Once Triggered:** There is unanimous consensus that once a patient with severe AS develops symptoms (even subtle exertional dyspnea or decreased exercise tolerance) or clear LV dysfunction, **AVR should be performed promptly**. Both ACC and ESC designate this scenario as Class I (highest priority) for intervention TCTMD.COM TCTMD.COM. The old "wait for symptoms" strategy is no longer about *whether* to intervene, but ensuring symptoms are recognized early – because unmitigated severe AS has a dire prognosis after symptom onset (high mortality within 2–3 years if untreated). Exercise testing is recommended by both societies to detect occult symptoms in apparently asymptomatic patients, reinforcing that **the threshold for declaring a**

patient "symptomatic" should be low and objectively tested.

- **High-Risk Asymptomatic Patients Benefit from Early AVR:** There is agreement that certain high-risk features justify preemptive AVR. Patients with **"very severe" AS (exceptionally high gradients/velocity)** or those showing **signs of incipient cardiac strain** (falling EF, rising BNP, exercise-induced symptoms) are consistently identified as likely to benefit from early intervention PMC.NCBI.NLM.NIH.GOV PMC.NCBI.NLM.NIH.GOV. The trials and meta-analyses show that these patients are more prone to adverse events if managed conservatively, and **early AVR in this context reduces heart failure events, sudden symptomatic crashes, and likely improves longer-term outcomes** PUBMED.NCBI.NLM.NIH.GOV PUBMED.NCBI.NLM.NIH.GOV. Thus, guidelines concur on recommending or at least considering AVR in asymptomatic severe AS if any such risk markers are present, as long as surgical/TAVR risk is acceptable.

- **Safety of AVR (Surgery or TAVR) in Asymptomatic Patients:** A notable consensus from recent data is that performing AVR *before* symptom onset does not carry an excess risk – in fact, operative mortality in trials was extremely low (0–1.4%) PUBMED.NCBI.NLM.NIH.GOV PUBMED.NCBI.NLM.NIH.GOV, and rates of stroke or major complications were similar between early intervention and observation groups PUBMED.NCBI.NLM.NIH.GOV. Modern surgical techniques and careful patient selection result in excellent perioperative outcomes in asymptomatic patients (who often have fewer comorbidities than symptomatic patients). Similarly, TAVR in asymptomatic individuals has proven **safe and effective**, with trial data showing no increase in stroke or death vs surveillance CRF.ORG. This allays the historical concern of "unnecessary surgery risk" – when done in experienced centers, **the procedural risk is low enough that it should not be a barrier to early treatment in an otherwise appropriate patient**.

- **Progressive Irreversible Damage:** There is consensus that even without symptoms, severe AS can cause **subtle myocardial damage** (fibrosis, diastolic dysfunction) and other complications (e.g. new atrial fibrillation) over time. Early AVR can preserve cardiac function by relieving afterload sooner. EARLY TAVR showed significantly less decline in left ventricular/atrial function in the early intervention group CRF.ORG. Collectively, experts agree that **preventing prolonged pressure overload on the heart is beneficial**, and this underpins the rationale for early intervention in many cases – to *prevent the "waiting" patient from turning into a heart failure patient.* As one

investigator put it, "there's not a lot of reassurance in waiting" once AS is severe TCTMD.COM.

- **Patient-Centered Decision Making:** Guidelines emphasize involving the patient in discussions about early AVR. There is agreement that asymptomatic patients must be educated on the risks of their condition and the pros/cons of early intervention. Some patients may strongly prefer to "get it taken care of" to avoid living with the Sword of Damocles of sudden symptoms, while others may wish to defer surgery as long as possible. A shared decision-making approach, ideally with a Heart Team (cardiologist, surgeon, etc.), is a standard of care endorsed by all major guidelines REVESPCARDIOL.ORG REVESPCARDIOL.ORG.

Areas of Ongoing Debate

Despite much convergence, there are **key controversies and conflicting viewpoints** in the management of asymptomatic severe AS:

- **Magnitude of Mortality Benefit:** The most debated point is whether early intervention actually improves *survival*, or merely prevents non-fatal morbidity. The RECOVERY trial's dramatic survival benefit PUBMED.NCBI.NLM.NIH.GOV stands somewhat in contrast to AVATAR, EARLY TAVR, and EVOLVED, which showed no significant all-cause mortality difference over a few years PUBMED.NCBI.NLM.NIH.GOV TCTMD.COM. Meta-analyses restricted to RCT data have concluded that **a survival advantage of early AVR is not yet definitively proven** (pooled RCT mortality difference was not statistically significant) PUBMED.NCBI.NLM.NIH.GOV. Critics argue that until we see clear mortality reduction across broader groups, routine early intervention for all asymptomatic patients might be premature. On the other hand, proponents point out that with longer follow-up, mortality curves may diverge (many patients in the "watchful" arms eventually get AVR anyway, diluting differences), and that preventing even a few sudden cardiac deaths or fatal heart failure episodes is meaningful. This debate means guidelines still stop short of a blanket Class I for all asymptomatic severe AS – the indication remains selective (Class IIa) pending more evidence of mortality benefit.

- **Composite Endpoints vs Patient-Centered Outcomes:** Many trials use composites that include hospitalizations and need for AVR as endpoints. This has drawn debate because including "needing a valve intervention" in the outcome can make early-intervention strategies look superior (since, by definition, they avoid the scenario of a later urgent intervention). For example, in EARLY TAVR the primary composite difference was largely driven by the high number of patients in the surveillance arm who were hospitalized to undergo eventual AVR TCTMD.COM . Some argue this **"bakes in" a favorable outcome for the early therapy group**, since an elective early procedure prevents a later unplanned procedure. Detractors say what ultimately matters more are *irreversible* outcomes like death, heart failure, or stroke. Indeed, when focusing on death or disabling stroke alone, early TAVR showed no significant difference CRF.ORG . An editorial comment on the ESC guidelines noted that the evidence for early AVR is somewhat weak when one considers that "events" in studies often include the valve surgery itself as an event (which is frequent and skews composites) REVESPCARDIOL.ORG . Thus, the field debates how to weigh **avoiding urgent interventions** (important for quality of life and resource use) versus **truly altering the disease's natural history**. Most agree the reduction in HF hospitalizations and acute presentations is valuable, but the lack of a clear mortality reduction in some studies invites differing interpretations of how aggressive to be.

- **Optimal Patient Selection:** There is still no perfect formula for identifying which asymptomatic patients will benefit most from early AVR. Ongoing debate surrounds the use of **novel risk markers** like CMR fibrosis (as tested in EVOLVED) or exercise parameters. EVOLVED's neutral result suggests that even if fibrosis is present, early surgery did not improve short-term mortality TCTMD.COM , surprising some who assumed fibrosis would identify a high-risk subset. This raises questions: should we intervene based on fibrosis or biomarkers, or stick to more conventional triggers? Similarly, how high should BNP be to tip the scale? And what about asymptomatic patients with comorbidities that limit exercise – are they truly asymptomatic or just unable to exert? These nuances fuel ongoing research. There is also the question of age: a very elderly asymptomatic patient might become symptomatic in a year – should we operate now or wait, given competing risks? Conversely, a relatively young patient might technically be asymptomatic but could safely delay surgery a bit to maximize later valve durability – or do they risk sudden death by waiting? These individualized considerations mean there is still debate and no one-size-fits-all rule. Heart Teams must carefully interpret risk markers on a case-by-case

basis until more data (or perhaps predictive models) refine selection criteria.

- **TAVR vs SAVR in Asymptomatic Patients:** With the advent of TAVR, a debate has emerged on the preferred mode of early intervention. For an older patient, early TAVR offers a less invasive fix with quicker recovery – making the threshold to intervene lower. The EARLY TAVR trial suggests that in low-risk septuagenarians, TAVR can be performed preemptively with excellent outcomes CRF.ORG. However, in younger patients, some argue **valve durability** is a concern: performing a TAVR (bioprosthetic valve) in a 60-year-old asymptomatic patient could mean they'll need a re-intervention in their lifetime (transcatheter valves may degenerate in 10-15 years). Surgeons might favor SAVR in such a patient to implant a larger surgical prosthesis (better suited for future valve-in-valve if needed). Current guidelines navigate this by recommending SAVR for younger/low-risk patients and reserving TAVR for older/high-risk, even in asymptomatic scenarios TCTMD.COM REVESPCARDIOL.ORG. But as TAVR technology evolves, this line may blur. Another aspect is that asymptomatic patients may be less likely to accept the small stroke risk of either procedure – making TAVR's lower upfront risk appealing, but data on long-term neurological outcomes (micro strokes, etc.) are still being gathered. The **TAVR vs SAVR mode debate** in asymptomatic AS is essentially the same as in symptomatic AS, but with an added layer: if we are intervening earlier, we want to choose a strategy that **maximizes long-term benefit** and minimizes the need for re-intervention. This remains an area of active discussion and study.

- **Evidence Gaps and Ongoing Trials:** Lastly, experts acknowledge that we still lack large-scale, long-term data for early intervention. The trials completed thus far, while compelling, have relatively short follow-up for a disease that spans decades. Ongoing studies (like the EASY-AS trial in Australia/New Zealand, and long-term extensions of EARLY TAVR and EVOLVED) will continue to inform practice. There is debate on how to incorporate emerging evidence – for example, if EARLY TAVR shows no mortality benefit at 5 years but better quality of life, is that enough for guidelines to recommend TAVR for asymptomatic patients broadly? Or should we wait for 10-year outcomes (especially for valve durability)? Such questions reflect the cautious approach guidelines take, and opinions can differ among cardiologists.

Implications for Clinical Decision-Making

The evolving evidence has already **shaped a more proactive approach** to severe AS: there is a clear trend toward recommending **earlier AVR in patients who have severe stenosis *even before classic symptoms develop*, provided their surgical or procedural risk is low.** The rationale is that the downsides of waiting (sudden death, heart failure, irreversible LV remodeling, frailty from limited activity) often outweigh the now-low risk of an AVR procedure. For the practicing clinician, this means asymptomatic severe AS is no longer a "do nothing until syncope" scenario – it requires vigilant follow-up and a low threshold to refer for intervention when risk features emerge.

Key takeaways for practice include:

- **Rigorous Assessment of "Asymptomatic":** Patients with severe AS should be carefully evaluated for any subtle symptoms (fatigue, reduced exercise capacity) and ideally undergo exercise testing if they deny symptoms. Many "asymptomatic" patients will actually have exercise limitation if tested. Unmasking symptoms or blood pressure drop on exercise test *immediately* reclassifies the patient as symptomatic and an AVR candidate TCTMD.COM PMC.NCBI.NLM.NIH.GOV. Thus, truly asymptomatic severe AS should be a deliberate diagnosis, and if confirmed, these patients merit frequent monitoring and education about reporting symptoms.

- **Monitor Risk Markers:** Follow trend of echocardiographic measurements (gradient/velocity progression), BNP levels, and LV ejection fraction in asymptomatic severe AS. The development of any high-risk marker (for example, a rise in gradient or a drop in EF even to ~50%) should prompt consideration of moving forward with AVR before waiting further REVESPCARDIOL.ORG. Advanced imaging like strain echo or cardiac MRI may help in special cases (e.g. detecting fibrosis or latent dysfunction), but their exact role is still being clarified (EVOLVED suggests MRI fibrosis alone shouldn't automatically trigger AVR without other indications).

- **Heart Team and Patient Discussion:** When considering early intervention, involve a multidisciplinary Heart Team (including a cardiothoracic surgeon and interventional cardiologist) to determine the optimal type of AVR and timing. Discuss with the patient the evidence: explain that while they feel well now, severe AS is a ticking clock that can cause sudden problems, and trials show intervening early can **prevent serious events and likely improve long-term health** CRF.ORG PUBMED.NCBI.NLM.NIH.GOV. Also discuss the small but real risks of the procedure and the fact that some patients do just fine for a while without surgery – it's a balance of risks. This shared decision process is crucial, as patient preference matters in asymptomatic situations.

- **Choice of SAVR vs TAVR:** Use guideline recommendations based on age and surgical risk to choose the intervention mode, but individualize it. For example, an otherwise healthy 60-year-old with asymptomatic severe AS and very high gradient might be advised to get a surgical AVR now (with a low operative risk and durable result) rather than wait to become symptomatic. In contrast, an 80-year-old asymptomatic patient might be an ideal candidate for early TAVR, avoiding the risk that they deteriorate to NYHA III heart failure in the next year TCTMD.COM. As new data (like long-term TAVR durability in low-risk patients) emerges, these recommendations will continue to be refined.

- **Continued Surveillance if Deferring:** If a decision is made to continue observation in an asymptomatic severe AS patient (for instance, no risk markers and patient prefers to wait), this is not a "static" decision. **Close follow-up (e.g. exams and echocardiography every 6 months)** is required, and the patient should be instructed to promptly report any change in exercise tolerance, chest pain, or dizziness. Essentially, the window of truly safe observation may be limited – many such patients will likely come to AVR within a year or two as data shows TCTMD.COM. The clinician should be ready to pivot to intervention at the earliest sign of change.

In conclusion, the management of severe asymptomatic aortic stenosis has shifted towards **earlier, preemptive intervention in many cases**, driven by evidence from recent RCTs and observational studies. Early AVR (whether via SAVR or TAVR) has been shown to **reduce heart failure events, prevent sudden symptom crashes, and preserve cardiac function**, with very low procedural risk

CRF.ORG PUBMED.NCBI.NLM.NIH.GOV. These benefits come without a clear immediate mortality penalty, and possibly a long-term survival advantage for certain patients PUBMED.NCBI.NLM.NIH.GOV. As a result, current guidelines (ACC/AHA, ESC) now endorse early AVR for asymptomatic patients who have any high-risk features, and they universally recommend not waiting if there is any objective evidence of symptom onset TCTMD.COM REVESPCARDIOL.ORG. Areas of debate remain – particularly regarding how to identify the optimal timing for those who truly have no symptoms and no red flags, and how to balance the longevity of prosthetic valves against the urgency of intervention. The field is rapidly evolving: ongoing studies and longer-term follow-up will likely further inform the risk-benefit calculus. For now, managing asymptomatic severe AS requires a nuanced, individualized approach, but the **general trend is clear**: “watchful waiting” is giving way to **“early fixing”** in order to avert the high costs of waiting for a crisis.

Sources:

- Kang DH et al. (RECOVERY Trial), *N Engl J Med.* 2020 – Early SAVR vs conservative care in very severe asymptomatic AS PUBMED.NCBI.NLM.NIH.GOV PUBMED.NCBI.NLM.NIH.GOV.
- Banovic M et al. (AVATAR Trial), *Circulation.* 2022 – Early SAVR vs conservative in asymptomatic severe AS PUBMED.NCBI.NLM.NIH.GOV PUBMED.NCBI.NLM.NIH.GOV.
- Généreux P et al. (EARLY TAVR Trial), TCT 2024 / *NEJM* 2024 – Early TAVR vs surveillance in asymptomatic severe AS CRF.ORG CRF.ORG.
- Loganath K et al. (EVOLVED Trial), *JAMA.* 2024 – Early AVR in asymptomatic AS with myocardial fibrosis TCTMD.COM TCTMD.COM.
- Costa GN et al., *Heart.* 2023 – Meta-analysis of early AVR vs watchful waiting (12 studies) PUBMED.NCBI.NLM.NIH.GOV PUBMED.NCBI.NLM.NIH.GOV.
- Senguttuvan NB et al., *Open Heart.* 2024 – Meta-analysis (RCTs and observational) of early AVR vs conservative PUBMED.NCBI.NLM.NIH.GOV PUBMED.NCBI.NLM.NIH.GOV.
- Otto CM et al. (ACC/AHA Valve Guidelines), *Circulation.* 2021 – Recommendations for asymptomatic severe AS TCTMD.COM TCTMD.COM.

- Vahanian A et al. (ESC/EACTS Valve Guidelines), *Eur Heart J.* 2022 – Recommendations for asymptomatic severe AS REVESPCARDIOL.ORG REVESPCARDIOL.ORG .
- TCTMD News October 2024 – "EARLY TAVR" and "EVOLVED" trial coverage TCTMD.COM TCTMD.COM .
- TCTMD News Sept 2021 – ESC guideline updates (age cutoffs, earlier intervention) TCTMD.COM REVESPCARDIOL.ORG .
- **Additional references:** ACC/AHA 2020 Guideline Key Points PMC.NCBI.NLM.NIH.GOV PMC.NCBI.NLM.NIH.GOV ; ESC 2021 Guideline Commentary (Rev Esp Cardiol) REVESPCARDIOL.ORG REVESPCARDIOL.ORG ; McKeown L. TCTMD 2024 (EARLY TAVR results) TCTMD.COM TCTMD.COM ; CRF/TCT 2024 Press Release (EARLY TAVR) CRF.ORG CRF.ORG .
