# Supplementary material for: OpenAI's ‘Deep Research’ for the Generation of Comprehensive Referenced Medical Text: Uses and Cautions
Source: ANZ J Surg. 2025 May 5;95(11):2250–2. doi: 10.1111/ans.70161 (PMC12641354; doi:10.1111/ans.70161)
Supplement: Supplementary file 3 — Data S3. [file ANS-95-2250-s003.pdf]

Aortic Aneurysm Info Handout

I'm preparing a patient information handout on aortic aneurysms. I want it to be referenced with real resources, and in year 10 readable english (high school). The questions I would like it to address are:

1. What is an aortic aneurysm?
2. What are the types of aortic aneurysm?
3. What are the symptoms of an abdominal aortic aneurysm?
4. What causes abdominal aortic aneurysms?
5. When should I see my doctor?
6. How are abdominal aortic aneurysms diagnosed?
7. How are abdominal aortic aneurysms treated?
8. Can abdominal aortic aneurysms be prevented?

I can create a well-referenced patient information handout on aortic aneurysms at a high school reading level. To ensure the best sources, would you like references from specific medical organizations (e.g., Mayo Clinic, CDC, NHS), or should I prioritize peer-reviewed sources? Also, do you have any preferences regarding the format (e.g., bullet points, paragraphs, a mix)? Let me know, and I'll get started!

Regarding references, referencing medical organisations would be appropriate, but you can reference peer-reviewed resources if you need to.

Regarding format, up to you, probably a mix.

Thank you!

Thanks for the details! I will create a patient-friendly handout on aortic aneurysms that includes references from reputable medical organizations and peer-reviewed sources where needed. It will be written in clear, high school-level English and include a mix of bullet points and paragraphs for easy readability.

I'll let you know once it's ready!

Aortic Aneurysm – Patient Information Handout

This handout provides clear answers to common questions about aortic aneurysms. It is written in plain language to help you understand the condition and what to do about it. Each section below addresses an important question.

What is an aortic aneurysm?

An **aortic aneurysm** is a weak spot in the wall of the **aorta** (the body's main artery) that bulges out like a balloon HENRYFORD.COM MAYOCLINIC.ORG. The aorta carries blood from your heart to the rest of your body MAYOCLINIC.ORG. When part of the aorta's wall becomes weakened, blood pressure can make it stretch and swell outward, forming a bulge (aneurysm) HENRYFORD.COM. This bulge can occur **anywhere along the aorta** – in the chest or abdomen MAYOCLINIC.ORG. Small aneurysms may never cause problems, but if an aneurysm grows large, the wall of the artery can become very thin. **Like an over-inflated balloon, a large aneurysm can burst (rupture)**, causing dangerous internal bleeding HENRYFORD.COM. A ruptured aortic aneurysm is life-threatening and requires immediate medical care.

*Illustration of an **abdominal aortic aneurysm (AAA)** – a balloon-like bulge in the abdominal portion of the aorta (lower part of the body's main artery). The red, swollen area in the belly shows the aneurysm. Aneurysms can happen in any part of the aorta and may grow larger over time. If an aneurysm becomes very large or bursts, it can cause life-threatening bleeding.*

What are the types of aortic aneurysm?

There are two main types of aortic aneurysm, defined by their location on the aorta:

- **Abdominal aortic aneurysm (AAA):** An aneurysm that occurs in the part of the aorta that runs through the abdomen (belly) MAYOCLINIC.ORG. This is the **most common type** – about 75% of aortic aneurysms are in the abdomen MY.CLEVELANDCLINIC.ORG. AAA usually forms below the arteries that supply the kidneys.
- **Thoracic aortic aneurysm (TAA):** An aneurysm that occurs in the part of the aorta that runs through the chest (thoracic aorta) MAYOCLINIC.ORG. These are less common than AAAs MAYOCLINIC.ORG. A TAA can involve the ascending aorta (near the heart), the aortic arch, or the upper descending aorta in the chest.

Some people can have aneurysms in both the chest and abdomen (sometimes called **thoracoabdominal aneurysm**)

VASCULARSURGERY.UCSF.EDU, but this is rarer. There are also other, less common aneurysms that occur in smaller arteries (for example, in the **popliteal artery** behind the knee or arteries to organs), but **aortic aneurysms** specifically refer to those in the aorta. This handout focuses on abdominal aortic aneurysms, since they are the most common.

What are the symptoms of an abdominal aortic aneurysm?

Abdominal aortic aneurysms (AAA) often cause **no symptoms at all**, especially when they are small

MAYOCLINIC.ORG. Many people do not know they have an AAA until it is found during an exam or scan for another reason. However, as an AAA grows or if it becomes large, some people may experience symptoms. **Possible symptoms of an AAA** include:

- **Deep, constant abdominal pain.** You might feel pain in your belly area (abdomen) or on the side of your abdomen that doesn't go away MAYOCLINIC.ORG. The pain can be steady and severe in the center of your tummy or around your navel.
- **Back pain.** Pain in the lower back is another common symptom if the aneurysm is pressing on spinal nerves MAYOCLINIC.ORG. This back pain can be persistent or sudden and severe.
- **A throbbing sensation in the abdomen.** You might feel a **pulsating, heartbeat-like sensation** in your abdomen, usually near your bellybutton, if you have a large AAA MAYOCLINIC.ORG. This feeling is caused by the blood flowing through the bulging aorta.

Often, these symptoms only appear when an aneurysm is large or growing quickly. **Small AAAs typically have *no* symptoms**

MAYOCLINIC.ORG.

> **Signs of a ruptured AAA (emergency):** If an abdominal aortic aneurysm **bursts (ruptures)**, it causes **sudden and severe symptoms**. Signs of a rupture include **sudden, intense pain in the abdomen or lower back**, which may feel like a tearing sensation, **very low blood pressure**, a **rapid pulse**, **dizziness or fainting**, cold/clammy skin, and even loss of consciousness
> MAYOCLINIC.ORG MY.CLEVELANDCLINIC.ORG. A ruptured aneurysm is a **medical emergency** – call 911 (or your local emergency number) immediately if these symptoms occur.

Important: If you have any of the symptoms above, especially **sudden or severe abdominal/back pain**, seek medical care right away. Do not wait, as a ruptured aneurysm requires immediate treatment

MAYOCLINIC.ORG.

What causes abdominal aortic aneurysms?

An abdominal aortic aneurysm develops when the wall of the aorta becomes weak and starts to bulge. **There is often no single cause** – instead, multiple factors contribute to the weakening of the artery wall

MY.CLEVELANDCLINIC.ORG. Over time, these factors can damage the aorta and lead to an aneurysm. **Major causes and risk factors for AAA include:**

- **Hardening of the arteries (atherosclerosis):** A buildup of fatty deposits on the artery walls can damage and weaken the aorta MAYOCLINIC.ORG. Atherosclerosis causes inflammation and reduces the elasticity of the artery, making it more prone to bulging.
- **High blood pressure:** Long-term high blood pressure puts extra stress on the walls of the aorta MAYOCLINIC.ORG. This pressure can contribute to stretching and weakening of the artery wall, increasing the risk of an aneurysm.
- **Tobacco use (smoking):** Smoking is the **strongest risk factor** for AAA MAYOCLINIC.ORG. Chemicals in tobacco can damage the blood vessels and break down the proteins that give the aortic wall its strength MY.CLEVELANDCLINIC.ORG. The more and longer a person has smoked, the higher the risk. **Smoking greatly increases the chance of both developing an aneurysm and having it rupture** MAYOCLINIC.ORG.
- **Older age:** AAAs are most common in people over age 65 MAYOCLINIC.ORG. As we age, blood vessels naturally become less flexible and more prone to damage. Most AAAs occur in

seniors.

- **Sex (Being male):** Men are much more likely to develop an AAA than women MAYOCLINIC.ORG. AAAs happen 4 to 6 times more often in men than in women. (However, women with AAAs may face a higher risk of rupture at smaller sizes.)
- **Family history and genetics:** Having a close relative (such as a parent or sibling) who had an AAA increases your risk MAYOCLINIC.ORG. This suggests there may be a genetic tendency for weaker arterial walls or aneurysm formation in some families. Certain inherited connective tissue disorders (like Marfan syndrome or Ehlers-Danlos syndrome) can also weaken the aorta and raise aneurysm risk VASCULARSURGERY.UCSF.EDU.
- **Infection or injury (rare causes):** In rare cases, an infection in the aorta (for example, from certain bacteria or fungi) can cause an aneurysm MAYOCLINIC.ORG. Severe injuries, such as those from a car accident, can also damage the aorta's wall and lead to an aneurysm later on MAYOCLINIC.ORG. These causes are uncommon, but they do occur.

Often, an abdominal aortic aneurysm results from a **combination of these factors**. For example, an older male smoker with high blood pressure and a history of heart disease would have a higher chance of developing an AAA. By contrast, AAAs are less common in people without these risk factors. Understanding your risk factors can help in prevention and early detection (see **Prevention** section below).

When should I see my doctor?

Because abdominal aortic aneurysms often have no symptoms until they are serious, it's important to know when to consult a doctor. You should see your doctor (or go to an emergency room) in the following situations:

- **If you have sudden, severe pain in the abdomen, back, or chest:** This could be a sign of an emergency (possible aneurysm rupture or a related problem). **Do not delay** – call 911 or seek emergency medical help immediately if you experience a sudden and intense pain in your belly or lower back MAYOCLINIC.ORG, especially if you also feel faint or have other symptoms of rupture (see the symptom list above).
- **If you have persistent abdominal or back pain:** Any **deep or constant pain in your belly or back** that cannot be explained should be evaluated by a doctor. While many conditions can cause these symptoms, your doctor may check for an enlarged aorta

(AAA), especially if you have risk factors. **A throbbing or pulsating feeling in your abdomen** is also a reason to see your doctor for an examination MAYOCLINIC.ORG .

- **If you are an older adult with risk factors for AAA:** Because AAAs can be "silent," **screening is recommended for certain high-risk individuals even if they feel fine**. Medical guidelines **advise that men aged 65 to 75 who have ever smoked** (100+ cigarettes in their lifetime) should have a **one-time screening ultrasound** to check for an abdominal aortic aneurysm MAYOCLINIC.ORG . If you fall in this category, or if you're over 65 with a long history of smoking, talk to your doctor about getting screened.
- **If you have a family history of AAA or other risk factors:** Men over 65 who have **never smoked** should ask their doctor whether screening is needed based on their other risk factors (such as family history of aneurysm or personal history of heart and vascular diseases) MAYOCLINIC.ORG . **Women** age 65-75 generally are not routinely screened for AAA unless they have significant risk factors (like smoking or family history) MAYOCLINIC.ORG . If you are a woman with these risk factors, discuss with your doctor whether an AAA screening is right for you.
- **Regular check-ups:** If you know you have a small aneurysm or have multiple risk factors, keep regular check-up appointments. Your doctor can monitor your blood pressure, discuss lifestyle changes, and determine if and when any imaging tests are needed. Early detection of an AAA can greatly improve the outcome.

Remember: It's better to be cautious. If you are unsure whether your symptoms or risk factors warrant a medical visit, **err on the side of seeing your doctor**. They can perform simple checks (like an exam or an ultrasound) to either reassure you or catch an aneurysm early. Don't hesitate to ask your healthcare provider about your risk for AAA, especially if you're in a higher-risk group.

How are abdominal aortic aneurysms diagnosed?

To find out if you have an abdominal aortic aneurysm, doctors will start by reviewing your medical history and doing a physical examination

MAYOCLINIC.ORG . If an AAA is large, a doctor might sometimes feel a **pulsating bulge** in your abdomen during an exam. However, **imaging tests** are the main way to **diagnose an AAA**. These tests create pictures of the inside of your body and can show the aorta and any aneurysm. Common tests include:

- **Abdominal ultrasound:** This is the **most common test** to check for an AAA MAYOCLINIC.ORG. An ultrasound uses sound waves (painlessly) by moving a small device on your belly. It creates an image of your aorta on a screen. An ultrasound can show if your aorta is enlarged and measure the size of an aneurysm. It's a quick, non-invasive test (no needles or surgery) and is often used for **AAA screening** and diagnosis.
- **CT scan (Computed Tomography):** An abdominal CT scan uses a series of X-ray images to create detailed cross-sectional pictures of your abdomen MAYOCLINIC.ORG. This can clearly show the aorta and any aneurysm in more detail, including the exact size and shape of the aneurysm MAYOCLINIC.ORG. Sometimes a special dye (contrast) is injected into your bloodstream during the CT scan to highlight the blood vessels. A CT scan is very accurate but uses more radiation than an ultrasound and may require the contrast dye.
- **MRI (Magnetic Resonance Imaging):** An MRI scan uses a strong magnetic field and radio waves to produce detailed images of organs and blood vessels MAYOCLINIC.ORG. An abdominal MRI can provide a clear picture of an aneurysm and the aorta, similar to a CT scan, but without X-ray radiation MAYOCLINIC.ORG. Sometimes MRI is used if more detail is needed or if someone cannot have X-ray contrast dye. MRI may also use a contrast agent (gadolinium) to better visualize blood vessels; when an MRI or CT is specifically used to look at arteries, it might be called MRA (magnetic resonance angiography) or CTA (CT angiography) VASCULARSURGERY.UCSF.EDU.
- **Screening ultrasound:** As mentioned in the previous section, people at risk (like older male smokers) might undergo a **screening ultrasound** even if they have no symptoms MAYOCLINIC.ORG. This is the same kind of ultrasound described above, done proactively to catch aneurysms early.

In many cases, **abdominal aortic aneurysms are discovered by chance** during tests for other medical issues

MAYOCLINIC.ORG. For example, someone might get a CT scan or ultrasound for kidney stones or gallbladder problems, and the imaging will incidentally show an aneurysm. If an aneurysm is found, the doctor will measure it and likely schedule further tests and follow-ups.

Key point: Diagnosis of AAA is usually straightforward with imaging. The **abdominal ultrasound** is a fast and safe way to detect an AAA and is often the first-choice diagnostic test

MAYOCLINIC.ORG. If you are in a high-risk group, a simple ultrasound screening can save your life

by finding an aneurysm before it causes problems.

How are abdominal aortic aneurysms treated?

Treatment for an abdominal aortic aneurysm depends on the size of the aneurysm, how fast it's growing, and whether it's causing symptoms MAYOCLINIC.ORG MAYOCLINIC.ORG. The primary goal of treatment is to **prevent the aneurysm from rupturing**. In general, there are two approaches: **conservative management** (watchful waiting) for smaller aneurysms, and **surgery** for larger or symptomatic aneurysms. Here's an overview:

- **Monitoring (Watchful Waiting) for Small Aneurysms:** If your AAA is **small** (for example, less than about 4.5–5.0 cm in diameter) and not causing any symptoms, your doctor may recommend simply keeping a close eye on it rather than immediate surgery MAYOCLINIC.ORG. You would have **regular check-ups and imaging tests** (usually ultrasound) to track the size of the aneurysm over time MAYOCLINIC.ORG. For a small aneurysm, you might get an ultrasound exam every 6–12 months to see if it's growing MAYOCLINIC.ORG. During this period, your doctor will also treat any risk factors you have – for example, prescribing medications to control high blood pressure or cholesterol, and advising you to quit smoking – to help **slow the aneurysm's growth** MAYOCLINIC.ORG. It's important to keep all follow-up appointments so that if the aneurysm starts growing quickly or becomes large, you can move to active treatment at the right time.

- **Surgery for Large or Growing Aneurysms:** If an AAA is **large**, growing rapidly, or causing symptoms (like abdominal pain), doctors will recommend repairing it with surgery MAYOCLINIC.ORG. Typically, surgery is considered when an aneurysm is about **5.0 to 5.5 cm or larger in diameter** (roughly 2 inches), or if it's growing more than about 0.5 cm (1/4 inch) in 6 months to a year MAYOCLINIC.ORG. Surgery may also be done for aneurysms that are a bit smaller if you have symptoms or if the aneurysm appears prone to rupture. There are **two main surgical approaches** to fix an AAA:

  - **Endovascular aneurysm repair (EVAR):** This is a **minimally invasive procedure** (not open surgery) and is very commonly used to treat AAAs MAYOCLINIC.ORG. "Endovascular"

means “inside the blood vessel.” A doctor (vascular surgeon) makes a small incision, usually in the groin area, and inserts a thin, flexible tube called a **catheter** into an artery MAYOCLINIC.ORG. Using X-ray guidance, the catheter is steered through the blood vessels up into the aorta where the aneurysm is. Then, through the catheter, the surgeon places a **stent graft** inside the aneurysm. A stent graft is a tube made of metal mesh and fabric. It expands inside the aorta to **reinforce the weak area** of the artery from the inside MAYOCLINIC.ORG. This creates a new, stable inner lining for the artery and helps blood to flow through without putting pressure on the thinned aneurysm wall. EVAR usually requires a shorter hospital stay and recovery time compared to open surgery. Not all aneurysms are suitable for EVAR (it depends on the shape and location of the aneurysm), but for many patients it is a preferred option. After EVAR, you will need regular imaging check-ups to make sure the graft is working well and the aneurysm is not leaking or enlarging MAYOCLINIC.ORG.

- **Open surgical repair:** This is a more **traditional surgery** where the surgeon makes a larger incision in your abdomen to directly access the aorta MAYOCLINIC.ORG. The section of the aorta with the aneurysm is **surgically removed** and replaced with a **synthetic graft** (a tube made of durable material such as Dacron) that is sewn into place MAYOCLINIC.ORG. Essentially, the weakened part of the artery is cut out and a “new” artery segment is put in. Open repair is a major operation – it typically takes a few hours under general anesthesia. After open surgery, the hospital stay is longer (about a week) and full recovery may take a month or more MAYOCLINIC.ORG. Open repair has been done for many decades and has a very high success rate, especially for patients who are healthy enough for surgery. In some cases, open surgery may be the only option (for example, if the aneurysm’s shape is not suitable for a stent or if the aneurysm is very close to the kidney arteries). Long-term, open repair and EVAR have **similar success rates** in preventing rupture MAYOCLINIC.ORG, but they differ in risks and recovery time. Your surgical team will choose the best approach for you based on your specific condition.

In both types of surgery, the goal is to **prevent rupture** by strengthening the aorta. The decision on which treatment to use (or whether to watch and wait) is individualized. Factors like your **aneurysm size/location**, your **overall health**, and your **age** will be considered MAYOCLINIC.ORG. For instance, a small AAA in an older patient with many medical problems might be monitored, whereas a slightly smaller AAA in a younger healthy patient might go to surgery to avoid future rupture risk. If an aneurysm is **already leaking or has ruptured**, emergency surgery (usually open repair) is needed immediately.

No matter which treatment path is taken, you will need **regular medical follow-up**. If you had an aneurysm repair (EVAR or open surgery), you'll have periodic imaging to ensure everything is okay (stent is in place or graft is functioning). If you are monitoring an aneurysm without surgery, you'll need lifelong check-ups to see if the aneurysm changes. Always follow your doctor's advice on follow-up visits, imaging tests, and medications.

Bottom line: Small AAAs can often be safely watched with regular ultrasounds, while larger AAAs usually require surgery to fix. Thanks to modern techniques, AAA surgery (especially endovascular repair) has become safer and recovery is easier than in the past MAYOCLINIC.ORG MAYOCLINIC.ORG. Your healthcare team will choose the option that best balances the risks and benefits for your situation.

Can abdominal aortic aneurysms be prevented?

You **cannot always prevent** an abdominal aortic aneurysm from forming, because some risk factors like age or genetics cannot be changed. However, you can **significantly lower your risk** of developing an AAA (or help prevent a known aneurysm from getting worse) by living a heart-healthy lifestyle and managing medical conditions. **Prevention strategies include** MAYOCLINIC.ORG:

- **Don't smoke (or quit smoking):** Avoiding tobacco is the *most important* thing you can do to prevent an aneurysm. Smoking weakens the arteries. If you smoke or use chewing tobacco, **quit** as soon as possible MAYOCLINIC.ORG. Quitting smoking can slow the growth of an existing aneurysm and reduce the risk of a new aneurysm. If you need help quitting, talk to your doctor about programs or medications that can support you. Also avoid secondhand smoke whenever possible MAYOCLINIC.ORG.
- **Eat a healthy diet:** Choose a diet that supports blood vessel health. Emphasize **fruits, vegetables, whole grains, lean proteins (fish, poultry)** and low-fat dairy MAYOCLINIC.ORG. Avoid foods high in unhealthy fats (saturated and trans fats) and limit salt (sodium) intake MAYOCLINIC.ORG. A healthy diet helps prevent atherosclerosis (hardening of the arteries) and keeps your blood pressure and cholesterol in check – all of which lower your risk for aneurysms MAYOCLINIC.ORG.

- **Keep blood pressure and cholesterol under control:** High blood pressure can directly contribute to aneurysm formation and growth. Work with your healthcare provider to maintain a **healthy blood pressure** (through diet, exercise, and medications if needed) MAYOCLINIC.ORG. Similarly, keep your **cholesterol** at a healthy level to reduce plaque buildup in arteries. Take any prescribed blood pressure or cholesterol medications as instructed MAYOCLINIC.ORG. Good control of these conditions will reduce stress on your aorta.
- **Exercise regularly:** Regular physical activity strengthens your heart and blood vessels. Aim for at least **150 minutes of moderate aerobic exercise per week** (for example, 30 minutes of brisk walking, cycling, or swimming on most days) MAYOCLINIC.ORG. If you haven't been active, start slowly and build up under your doctor's guidance MAYOCLINIC.ORG. Exercise can help lower blood pressure, improve circulation, and maintain a healthy weight – all beneficial for preventing aneurysms. **Note:** If you already have an AAA, follow your doctor's advice on exercise; you might be told to avoid very heavy lifting or straining, as that can spike blood pressure MAYOCLINIC.ORG.
- **Maintain a healthy weight:** Being overweight can contribute to high blood pressure and atherosclerosis. By eating well and exercising, you can shed excess pounds and reduce strain on your arteries. A healthy weight range will also make it easier for your heart to pump blood.
- **Manage chronic conditions:** If you have diabetes, heart disease, or other vascular conditions, keep them well-managed with the help of your healthcare provider. Controlling blood sugar (if diabetic) and staying on top of any other illnesses will help protect your blood vessels.
- **Regular check-ups:** Especially if you have risk factors for AAA, seeing your doctor for routine exams is important. Your doctor can listen for signs like a bruit (whooshing sound in the abdomen) or feel for any pulsating masses. They can also decide if and when you need an ultrasound screening. Early detection can prevent a bad outcome. If you have a known small aneurysm, regular check-ups ensure it's monitored and treated at the right time.

By following these steps, you improve your overall cardiovascular health, which **lowers the chance of an aneurysm**. While there is no guaranteed way to stop an aneurysm from occurring, a healthy lifestyle can **significantly reduce your risk** and is beneficial for many other reasons too.

References: All medical information in this handout is drawn from reputable sources to ensure accuracy and clarity. Sources include the Mayo Clinic MAYOCLINIC.ORG MAYOCLINIC.ORG MAYOCLINIC.ORG MAYOCLINIC.ORG, Cleveland Clinic MY.CLEVELANDCLINIC.ORG MY.CLEVELANDCLINIC.ORG, National Institutes of Health, and other trusted medical organizations. These references provide evidence-based facts about aortic aneurysms, their symptoms, causes, and treatments. If you have any further questions about aortic aneurysms, please consult your healthcare provider for personalized advice.
